# Honeycomb-inspired porous biomimetic scaffold with specific adaptability to host cells behavior for bone repair

**DOI:** 10.1186/s12951-026-04133-7

**Published:** 2026-02-13

**Authors:** Langjie Chai, Danchi Liu, Jie Chen, Shilin Jiang, Ye Lu, Lei Yu, Lu Zhang, Tonghe Zhu, Chao Liu, Chenglin Yang, Chengyuan Zhang, Huitang Xia, Dahang Zhao, Feng Yuan

**Affiliations:** 1 Department of Sports Medicine, Department of Orthopedics, Shanghai Sixth People’s Hospital, Shanghai Jiao Tong University School of Medicine, 600 Yishan Rd, Shanghai, 200233 P.R. China; 2 Department of Plastic Surgery, The First Affiliated Hospital of Shandong, First Medical University & Shandong Provincial Qianfoshan Hospital, 16766 Jingshi Rd, Jinan, 250014 Shandong P.R. China; 3 Jinan Clinical Research Center for Tissue Engineering Skin Regeneration and Wound Repair, Jinan, 250014 Shandong P. R. China; 4 Institute for Frontier Medical Technology, School of Chemistry and Chemical Engineering, Shanghai University of Engineering Science, 333 Longteng Rd, Shanghai, 201620 P.R. China; 5 Department of Orthopedics, Shanghai Public Health Clinical Center (Fudan University, 2901 Caolang Rd, Shanghai, 201500 P.R. China; 6 Shanghai Pengguan Biomedical Technology Co., Ltd, 2 Rd., Xinchang Town, Pudong New Area, Shanghai, 200120 P.R. China; 7 Department of Orthopaedics, Ruijin Hospital, Shanghai Jiaotong University School of Medicine, 197 Ruijin 2 Rd, Shanghai, 200000 P.R. China

**Keywords:** Biomimetic scaffold, Multiscale porous, Layered double hydroxides, Deferoxamine, Nano-bio interface, Vascularized bone regeneration

## Abstract

In the field of bone defect repair, the three-dimensional architecture and bioactivity of tissue-engineered bone scaffolds play a pivotal role. However, two major challenges remain: the cell type–specific response of host cells to scaffold pore architecture and the sustained promotion of angiogenesis and osteogenesis essential for large-segment bone regeneration. Inspired by the structural characteristics of natural honeycombs, this study integrates 3D printing with hydrogel engineering to construct a novel composite scaffold. Its macro-level innovation lies in a multilevel pore structure, while its functional core stems from a meticulously designed “nano-bio interface”: layered double hydroxides (LDHs) loaded with deferoxamine (DFO) are assembled into nanosheets (DFO@LDHs) as key functional units, and are simultaneously integrated into both a polycaprolactone (PCL) framework (D@LP) and a glycidyl methacrylate-modified hyaluronic acid (HA-GMA) hydrogel (D@LG) to form the final composite scaffold (D@LG/D@LP). This nano-bio interface not only enables sustained, controlled release of bioactive factors but also actively regulates cellular spatiotemporal behavior. The unique pore distribution and degradation characteristics of the hydrogel and 3D-printed scaffold preferentially guide endothelial cell adhesion and microvascular network formation, subsequently initiating stem cell differentiation and bone matrix deposition. Both in vitro and in vivo experiments confirm the scaffold’s excellent biocompatibility. Through a nanomaterial-driven cascade regulation mechanism, it coordinates the activation of angiogenesis and osteogenesis, significantly accelerating vascularized bone regeneration. This work demonstrates an effective strategy for empowering three-dimensional scaffolds through nano-bio interface construction, achieving spatiotemporal programming for complex tissue regeneration.

**Graphical Abstract:**

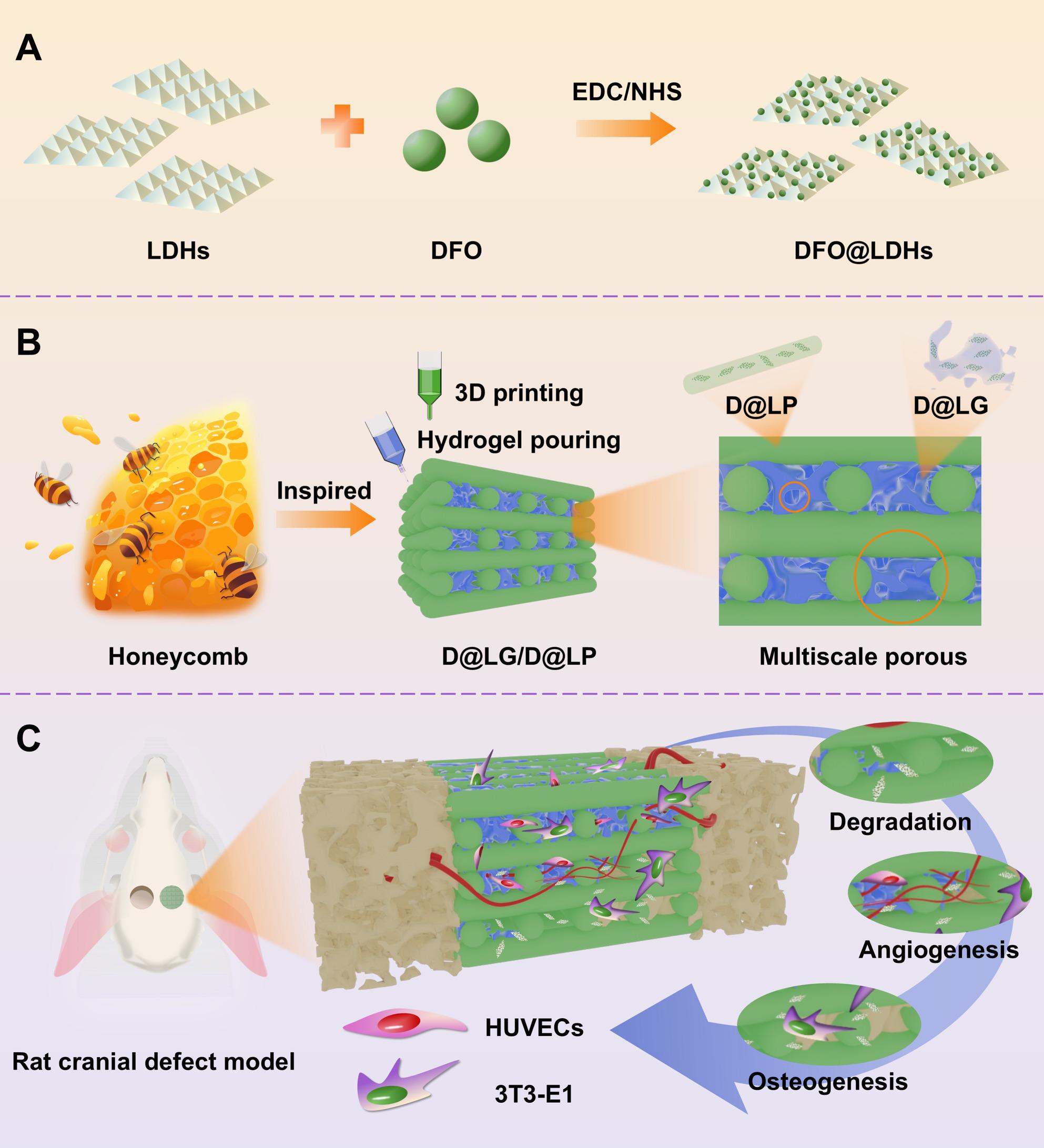

**Supplementary Information:**

The online version contains supplementary material available at 10.1186/s12951-026-04133-7.

## Introduction

Bone defects are commonly caused by trauma, infection, and tumors [1]. Although bone tissue possesses a certain degree of self-repair capability through the biological activities of bone-related cells, this regenerative capacity is inherently limited [2]. As a result, the repair of large-segment bone defects remains a significant clinical challenge. Autologous bone grafting, currently regarded as the gold standard for bone defect treatment, faces limitations in widespread application due to complications such as donor site morbidity, risk of infection, limited availability, and prolonged surgical time [3]. In response, research has increasingly focused on developing bone tissue engineering materials that combine an optimal three-dimensional architecture with robust bioactivity [4, 5]. While existing biomaterials primarily aim at basic bone regeneration, they often overlook the specific interactions between different host cell types and the scaffold structure. This absence of specific interactions leads to fundamental failure in bioconvergence. This manifests directly as impaired cell adhesion, restricted migration, and disrupted differentiation guidance, ultimately preventing the formation of three-dimensional tissue structures [6]. Therefore, the design and fabrication of bone scaffolds tailored to accommodate diverse host cellular responses hold great promise for advancing clinical outcomes in bone repair.

Various cell types demonstrate distinct sensitivities to scaffold pore size, a structural parameter that profoundly affects cellular biological behavior by modulating host cell proliferation, migration, and differentiation, thereby facilitating accelerated tissue regeneration [7–9]. Empirical evidence indicates that endothelial cells exhibit enhanced growth within scaffolds characterized by small pore sizes, with microvascular networks preferentially forming in pores measuring approximately 6 to 9 μm [10]. Conversely, larger pore sizes ranging from 125 to 350 μm are more conducive to bone tissue regeneration and the proliferation of smooth muscle cells associated with large blood vessels [11–14]. Consequently, the design of scaffolds for bone tissue engineering must not only ensure adequate mechanical strength but also optimize microstructural features, particularly pore size, to accommodate the specific biological requirements of the target cell populations, thereby effectively augmenting the regenerative capacity within bone defect sites.

Bone remodeling is fundamentally dependent on the tightly coordinated interplay between angiogenesis and osteogenic differentiation [15]. The establishment of an adequate and timely microvascular network within a scaffold plays a critical role in bone tissue development and regeneration by facilitating oxygen supply, nutrient transport, cellular recruitment, and the removal of metabolic waste [16, 17]. Deferoxamine (DFO), an iron chelator approved by the FDA for the treatment of transfusion-induced iron overload, has recently garnered significant interest in tissue engineering applications [18]. Empirical evidence indicates that DFO promotes the formation of “H-type” blood vessels through the stabilization of hypoxia-inducible factor-1α (HIF-1α) and the subsequent upregulation of vascular endothelial growth factor (VEGF), thereby synchronizing angiogenesis with osteogenesis and enhancing the recruitment and activation of bone-related cells [19, 20]. Nonetheless, the clinical utility of DFO is constrained by its high-water solubility and brief in vivo half-life, which result in rapid degradation and systemic dispersion upon direct administration, ultimately leading to diminished bioavailability and therapeutic efficacy [21]. Consequently, the development of controlled delivery systems capable of sustained and stable release of small molecule drugs is imperative to optimize the outcomes of bone tissue regeneration. Layered double hydroxides (LDHs) have emerged as promising materials for drug delivery systems owing to their high specific surface area, excellent biocompatibility, and superior drug-loading capabilities [22]. Additionally, LDHs have been demonstrated to significantly facilitate the differentiation of osteoprogenitor cells [23, 24]. When employed as carriers for DFO, LDHs not only enable sustained release of the drug, thereby enhancing local drug concentration and prolonging its therapeutic effect, but also synergistically exploit their intrinsic osteoinductive properties to further promote bone regeneration. Nonetheless, a critical challenge in bone tissue engineering remains the optimization of scaffold design to preserve favorable mechanical properties while integrating bioactivity and controlled drug release functionalities.

**Fig. 1:**
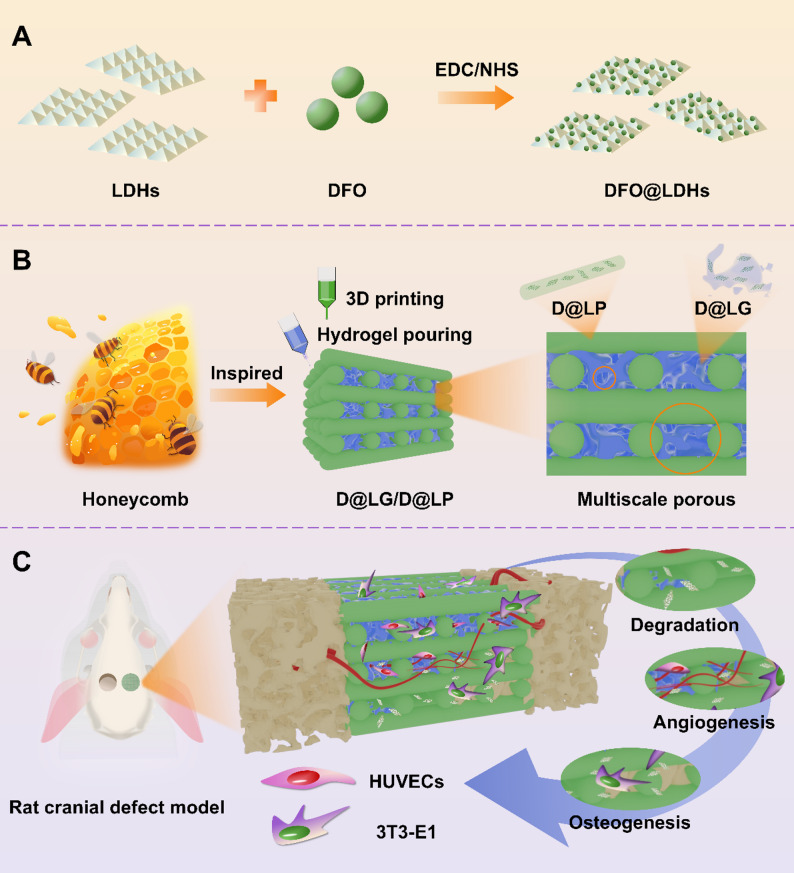
Schematic illustration of biomimetic D@LG/D@LP scaffold, which possesses the ability to continuously release DFO@LDH nanosheets and exhibits a multiscale porous structure. This scaffold dynamically regulates multiple spatiotemporally coordinated cellular responses, thereby promoting the regeneration process of vascularized bone tissue

The honeycomb structure, characterized by its robustness and porosity, serves primarily as a storage medium for honey and pollen. Drawing inspiration from this distinctive architecture, a tissue-engineered bone scaffold exhibiting hierarchical porosity was developed through the integration of hydrogels with a three-dimensional (3D) printed framework. DFO-loaded LDHs were synthesized into sheet-like nanosheets (DFO@LDHs) (Fig. 1A) and subsequently incorporated into a glycidyl methacrylate-modified hyaluronic acid (HA-GMA) hydrogel (DFO@LDHs/HA-GMA, denoted as D@LG) as well as into a 3D-printed polycaprolactone (PCL) scaffold (DFO@LDHs/PCL, denoted as D@LP). The D@LG hydrogel was then infused into the pores of the 3D-printed D@LP scaffold to fabricate a biomimetic composite scaffold (D@LG/D@LP) (Fig. 1B). Owing to the distinct pore dimensions and degradation kinetics of the hydrogel and the 3D-printed framework, the microporous architecture of the hydrogel promotes the formation of microvascular networks. It is important to note that this specific adaptability refers to the structural matching of pore dimensions to the biological requirements of different cell types (passive regulation), rather than an active stimuli-responsive mechanism. As the hydrogel preferentially degrades, a sustained release of DFO@LDH nanosheets is anticipated, thereby unveiling the larger pores within the 3D-printed framework to facilitate timely angiogenesis and stable osteogenesis. Concurrently, the slow degradation of the D@LP framework ensures long-term mechanical integrity throughout the bone regeneration process (Fig. 1C). Cellular responses, including proliferation, migration, angiogenesis, and osteogenic differentiation, were assessed using human umbilical vein endothelial cells (HUVECs) and mouse osteoblast precursor cells (3T3-E1) cultured on the biomimetic scaffold. Furthermore, a rat cranial defect model was employed to evaluate the scaffold’s in vivo vascularization and bone regeneration efficacy through micro-computed tomography (micro-CT) and histopathological analyses.

## Materials and methods

### Materials

DFO was purchased from Shanghai Yuanye Biotechnology Co., Ltd. Aluminum nitrate nonahydrate (Al(NO_3_)_3_·9H_2_O), magnesium nitrate hexahydrate (Mg(NO_3_)_2_·6H_2_O), sodium hydroxide (NaOH), and ethanol were purchased from Shanghai Chemical Co., Ltd. (3-aminopropyl) triethoxysilane (APTES) was provided by Aladdin Industries Co., Ltd. Hexafluoroisopropanol (HFIP, purity ≥ 99.2%) was from Shanghai Adamas Reagent Co., Ltd. L-ascorbic acid, glycerophosphate, and dexamethasone were purchased from Sigma. HUVECs and 3T3-E1 were collected from the Cell Bank of the Chinese Academy of Sciences. Bio-Tech Biotechnology Co., Ltd. (Shanghai) provided cell counting kit-8 (CCK-8), BCA protein assay kit, alkaline phosphatase colorimetric kit, alkaline phosphatase detection kit, and osteoblast mineralized nodule staining kit (Alizarin Red) for this study. The culture medium (DMEM) and fetal bovine serum (FBS) were purchased from Gibco Trading Co., Ltd. (Shanghai, China).

### Preparation of biomimetic scaffolds

#### Preparation of DFO@LDHs

##### Preparation of LDHs

LDHs were prepared by a hydrothermal method reported in the literature [25]. The detailed synthesis methods and processes of LDHs are provided in *Supporting Information 1.1*. The final product was freeze-dried at −80 ℃ under vacuum for 3 d to obtain LDHs powder.

##### Carboxylation of LDHs

100 mg LDHs were ultrasonically dispersed in 20 mL of 99% ethanol solution for 15 min. Under the protection of nitrogen, 100 µL APTES were added dropwise, condensed and refluxed at 70 ℃ for 8 h, then centrifugally separated, washed with ethanol for three times, and then freeze-dried and collected to obtain the white product LDHs-NH_2_, which were designated as A. A was ultrasonically dispersed in 20 mL absolute ethanol for 15 min to obtain solution (A) 0.049 g glutaric anhydride were mixed with 10mL absolute ethanol to obtain an ethanol solution of glutaric anhydride, which was recorded as solution (B) Under the protection of nitrogen, liquid B was added into liquid A drop by drop, then condensed and refluxed at room temperature for 6 h, centrifugally separated, washed with ethanol for three times, and the white product LDHs-COOH were collected, dried and preserved.

##### DFO grafted in carboxylated LDHs

Under the condition of ice bath, 50 mg of LDHs-COOH were ultrasonically dispersed in 10 mL of deionized water for 15 min, and 30 mg of NHS and 90 mg of EDC were sequentially added to activate carboxyl groups. Then, varying amounts of DFO (4, 6, 8, 10, and 12 mg) were added, stirred at high speed in the dark for 24 h, then centrifuged, washed with deionized water for three times, and the white product DFO@LDHs were collected.

#### Preparation of hydrogels

Acylated hyaluronic acid (HA-GMA) was prepared by the method reported in the literature [26]. LDHs or DFO@LDHs were dispersed in deionized water, respectively. After 15 min ultrasonic in an ice bath, 3% acylated hyaluronic acid were added in the above composites, following stir for 24 h in the dark. After they were stirred evenly, the obtained hydrogel mixture named LDHs/HA-GMA (LG) and DFO@LDHs/HA-GMA (D@LG), respectively.

#### Preparation of 3D printing ink and scaffolds

Disperse calculated amounts of LDHs or DFO@LDHs in hexafluoroisopropanol, followed by ultrasonic dispersion in an ice bath. After uniform dispersion, PCL was added and stirred in the dark for 48 h to achieve specific drug loading concentrations (detailed below). After PCL is completely dissolved and LDHs and DFO@LDHs are evenly dispersed, pour them into a mold and place it in a fume hood to wait for the hexafluoroisopropanol to evaporate, thereby obtaining LP and D@LP composites with varying DFO@LDHs contents.

The PCL scaffold (PCL), LDHs/PCL scaffold (LP), and DFO@LDHs/PCL scaffold (D@LP) were fabricated using a 3D bioprinter, respectively. Firstly, the uniformly cut PCL, LDHs/PCL, and DFO@LDHs/PCL flake particles were loaded into a stainless barrel, heated to 90 ℃ for 1 h, and extruded through a nozzle (0.3 mm) at 90 ℃ using a spiral mechanism at a constant extrusion rate of 0.01 mm^3^/s, respectively. The nozzle was moved at a speed of 1.5 mm/s during the printing process. The distance between microfilaments was controlled at 1 mm, and the scaffolds were constructed by depositing filaments with an angle of 90° and a thickness of 0.2 mm between consecutive layers. The scaffold finally assumes a cylindrical shape.

The injectable HA-GMA hydrogel (G), LDHs/HA-GMA hydrogel (LG), and DFO@LDHs/HA-GMA hydrogel (D@LG) were injected into the voids of the PCL, LP, and D@LP, respectively. After incubated for 1 h at 4 ℃, all the scaffolds were crosslinked using visible light, following freeze-dried for 48 h. Finally, in order to verify that the addition of HA-GMA hydrogel has the capacity to increase the biocompatibility of pure PCL scaffold, as well as the addition of DFO@LDHs has the potential to promote vascular osteogenesis to varying degrees, three hydrogel-enhanced composite scaffolds (HA-GMA hydrogel-enhanced PCL composite scaffold (G/P), LDHs/HA-GMA hydrogel-enhanced LDHs/PCL composite scaffold (LG/LP), and DFO@LDHs/HA-GMA hydrogel-enhanced LDHs/PCL composite scaffold (D@LG/D@LP) were prepared. Specifically, in order to determine the optimal concentration for loading DFO@LDHs into the scaffold, a series of DFO@LDHs concentrations (2 wt%, 1 wt%, 0.5 wt%, 0.25 wt%, and 0.125 wt%) were selected for the fabrication of D@LG/D@LP-2, D@LG/D@LP-1, D@LG/D@LP-0.5, D@LG/D@LP-0.25, and D@LG/D@LP-0.125 scaffolds, respectively.

### Characterization of LDHs and DFO@LDHs

X-ray photoelectron spectroscopy spectrum was obtained by X-ray photoelectron spectrometer (XPS, K-Alpha, Thermo Scientific, USA) in the scanning region of 0 ~ 1350 eV. An Ultima IV X-ray diffractometer (XRD, Rigaku Co., Tokyo, Japan) was adopted to characterize the crystallinity in the scanning region of 2θ = 5 ~ 100°. The morphology was observed by scanning electron microscope (SEM, KYKY Technology Development Co., Ltd., China). The functional groups of the sample were analyzed by potassium bromide on Fourier transform infrared spectrometer, and the range was 800 ~ 4000 cm^− 1^ (FTIR, Nicolet 6700, Thermo, USA). Zeta potentials were measured by zeta sizer nano instrument (ZETA, Malvern, Worcestershire, UK). Thermogravimetric analysis (TGA, TG209F1, Netzsch, Selb, Germany) were performed to determine the thermal properties of materials with the heating rate of 10 ℃/min in the scanning temperature region of room temperature to 800 ℃.

### DFO release test in vitro

In order to measure the drug loading efficiency of DFO in DFO@LDHs drug delivery system, the residual DFO drug content in supernatant was recorded by ultraviolet-visible spectrophotometer. DFO effectively chelates iron ions (Fe³⁺) to form stable complexes, which are detectable by UV-Vis spectrophotometry at a wavelength of 430 nm. In short, 100 µL of the collected solution was reacted with an equal volume of 3 mM FeCl_3_ solution for 10 min. Using the standard curve, the concentration of DFO in the supernatant was determined. The encapsulation efficiency (*EE*) and drug loading efficiency (*LE*) of DFO@LDHs were calculated using *(1)*, *(2)*, respectively:

1 $$\:EE\:\left(\%\right)=\frac{{W}_{A}-{W}_{R}}{{W}_{A}}\times\:100\%$$

2 $$\:LE\:\left(\%\right)=\frac{{W}_{A}-{W}_{R}}{{W}_{NPs}}\times\:100\%$$

Where *W*_*A*_, *W*_*R*_ and *W*_*NPs*_ represent total amount of added drug, the amount of remnant drug, and the amount of drug loaded LDHs, respectively.

Determination method of drug release curve: Firstly, 8 mg of DFO@LDHs was dispersed in a dialysis bag filled with 5 mL of PBS, and then transferred to 50 mL centrifugal tubes containing 20 mL of PBS, and then these tubes were shaken in a shaker at a speed of 100 r/min at 37 ℃. Supernatants were collected at different time points and replaced with fresh PBS of the same volume. Iron ions (Fe^3+^) were used to chelate DFO in supernatant, and the release concentration of DFO was determined by UV-Vis spectrophotometer. Finally, the release amount of DFO at each time point is analyzed and the release curve is drawn.

### Characterization of biomimetic scaffolds

Scanning electron microscope (SEM, KYKY Technology Development Co., Ltd., China) was used to observe the cross-sectional and longitudinal views of different scaffolds. The release of DFO from the scaffolds was determined as described in Sect. “DFO release test in vitro”, except that the scaffolds were placed in a dialysis bag containing 5 mL of PBS. The mass of the scaffolds during and after immersion was determined by the weight loss method. An Ultima IV X-ray diffractometer (XRD, Rigaku Co., Tokyo, Japan) was adopted to characterize the crystallinity in the scanning region of 2θ = 5 ~ 100°. Mercury intrusion porosimetry (MIP, Micromeritics AutoPore V 9620, USA) was used to determine the size and distribution of pores within the scaffolds. Micro in-situ mechanical testing machine (M-100, CARE Measurement & Control Co., Ltd., China) are used to evaluate the fatigue resistance of brackets. The details of water contact angle test and mechanical performance test are provided in *Supporting Information 1.2.and 1.3.*

### Bioactivity test and cytocompatibility evaluation

The specific methods of cell culture and preparation of osteogenic induction solution are provided in *Supporting Information 1.4.*

#### Cell proliferation test

PCL, G/P, LG/LP, and D@LG/D@LP were fumigated with 75% alcohol and sterilized by ultraviolet irradiation, then immersed in the medium (0.1 g/mL) to obtain the leaching solution, respectively. Human umbilical vein endothelial cells (HUVECs) were inoculated into 24-well plates, and the cell density was about 1 × 10^4^ cells per well. After the cells adhered to the wall, five groups (Control, PCL, G/P, LG/LP, D@LG/D@LP) of extraction media were added to the corresponding wells. In order to evaluate the cell proliferation ability, the cells were moistened with PBS at the predetermined time points of 1, 3, and 5 days of cell culture, and 0.5 mL CCK-8 solution prepared according to the manufacturer’s scheme was added to each well, then incubated at 37 ℃ for 60 min. Finally, the absorbance at 450 nm was recorded by an enzyme-labeled instrument.

In order to observe the proliferation of cells directly, cells were stained with Calcein-AM/PI, and observed and photographed by fluorescence microscope on the 3rd day. The cell proliferation of 3T3-E1 was detected, observed and photographed in the same way.

#### Angiogenesis-related test

In the experiment of scratch repair, HUVECs were inoculated into a 6-well plate at a density of 4 × 10^5^ cells/well. After the cells were 100% confluent, a clean and uniform scratch with no residual debris cells was drawn at the center line of the hole with a sterile 200 µL gun. Five groups (Control, PCL, G/P, LG/LP, D@LG/D@LP) containing 2% serum were added into the corresponding wells, and photographs were taken under the inverted phase contrast microscope at 0 h and 24 h respectively, and the number of cells migrating between the original scratch widths was measured under the microscope at 24 h.

In the tube-forming experiment, the matrix glue was melted in the refrigerator at 4 ℃ for several hours, and then 100 µL matrigel was added to a 24-well plate with a pipette and gelled at 37 ℃ for 30 min. Both the orifice plate and pipette are precooled at −20 ℃ in advance. After gelation, HUVECs were inoculated into a 24-well plate at a density of 4 × 10^5^ cells per well. After incubation for 20 h, samples were collected to capture images. Then, the number of junctions, the total length of pipes and the number of meshes were statistically analyzed by Image J.

#### Osteogenic differentiation, intervention, and ALP and ARS staining

2 × 10⁴ 3T3-E1 cells per well were seeded into 24-well plates, and osteogenic induction was initiated once the cells reached approximately 90% confluence. On the 14th day of induction, cells were stained with alkaline phosphatase chromogenic kit and imaged. On the 14th day of induction, the cells were washed with PBS and lysed with RIPA lysate, and the supernatant was collected after centrifugation. Alkaline phosphatase (ALP) activity was measured by alkaline phosphatase detection kit, and total protein content was evaluated by BCA protein detection kit. ALP activity in each sample was calculated according to the manufacturer’s instructions.

10 × 10^4^ 3T3-E1 cells per well were seeded into 6-well plates, and osteogenic induction was initiated once the cells reached approximately 90% confluence. On the 21 st day of induction, cells were stained with osteoblast mineralized nodule staining kit (alizarin red) and imaged, then 10% cetylpyridinium was added and incubated at room temperature for 30 min. Finally, the OD value was detected at 560 nm by enzyme-labeled instrument, and ARS was quantitatively determined by analyzing the OD value.

### Bone regeneration assessment in vivo

All rats were obtained from Shanghai Bikai Keji Biotechnology Co., Ltd. (Shanghai, China) and all animal experiments were approved by the Animal Welfare Ethics Committee of Shanghai Sixth People’s Hospital (ethical number: 2024 − 0176). Ethical principles were followed throughout the experiment. All experimental plans were proceeded in conformity of the Animal Management Regulations of China (1988 and revised in 2001, Ministry of Science and Technology).

Twenty Sprague-Dawley (SD) rats (8 weeks old, male, about 200 g) were randomly divided into four groups (*n* = 5): control group, G/P group, LG/LP group, and D@LG/D@LP group.

Their living conditions were maintained at 25 ℃ and 50% humidity, and the SD rats were fed with sufficient dry food and water to maintain health. A circular full-thickness defect with a diameter of 5 mm was created on the skull by surgery. Subsequently, cylindrical scaffolds (diameter: 5 mm) from the four groups were implanted into the defect site.

After 6 and 12 weeks of implantation, the rats were humanely euthanized, and their skulls were collected. The samples were scanned using a micro-CT system to evaluate the effects of different scaffolds on bone repair. Parameters such as bone mineral density (BMD), bone volume to tissue volume ratio (%, BV/TV), and so on were quantified and calculated.

After decalcification of all samples, tissue sections were obtained from the cross-sectional area at the center of each defect for histological evaluation using H&E staining and Masson’s trichrome staining to assess osteogenesis. To further investigate the angiogenic-osteogenic coupling effect, immunofluorescence staining was performed to detect osteogenic markers, including osteocalcin (OCN) and type I collagen (COL I), as well as the angiogenic marker platelet-endothelial cell adhesion molecule (CD31), followed by quantitative analysis.

### Statistics

Two groups were compared using the unpaired double-tailed student T-test. The Tukey ANOVA post hoc test was performed for one-way ANOVA. Sidak’s multiple comparison test was performed for two-way ANOVA. p value < 0.05 was considered statistically significant. ^*^p < 0.05, ^**^p < 0.01, ^***^p < 0.001, and “ns” indicated no significant difference. In all groups, data were analyzed from at least three separate experiments.

## Results and discussion

### Physicochemical properties of DFO@LDHs nanoparticles and DFO release properties

**Fig. 2:**
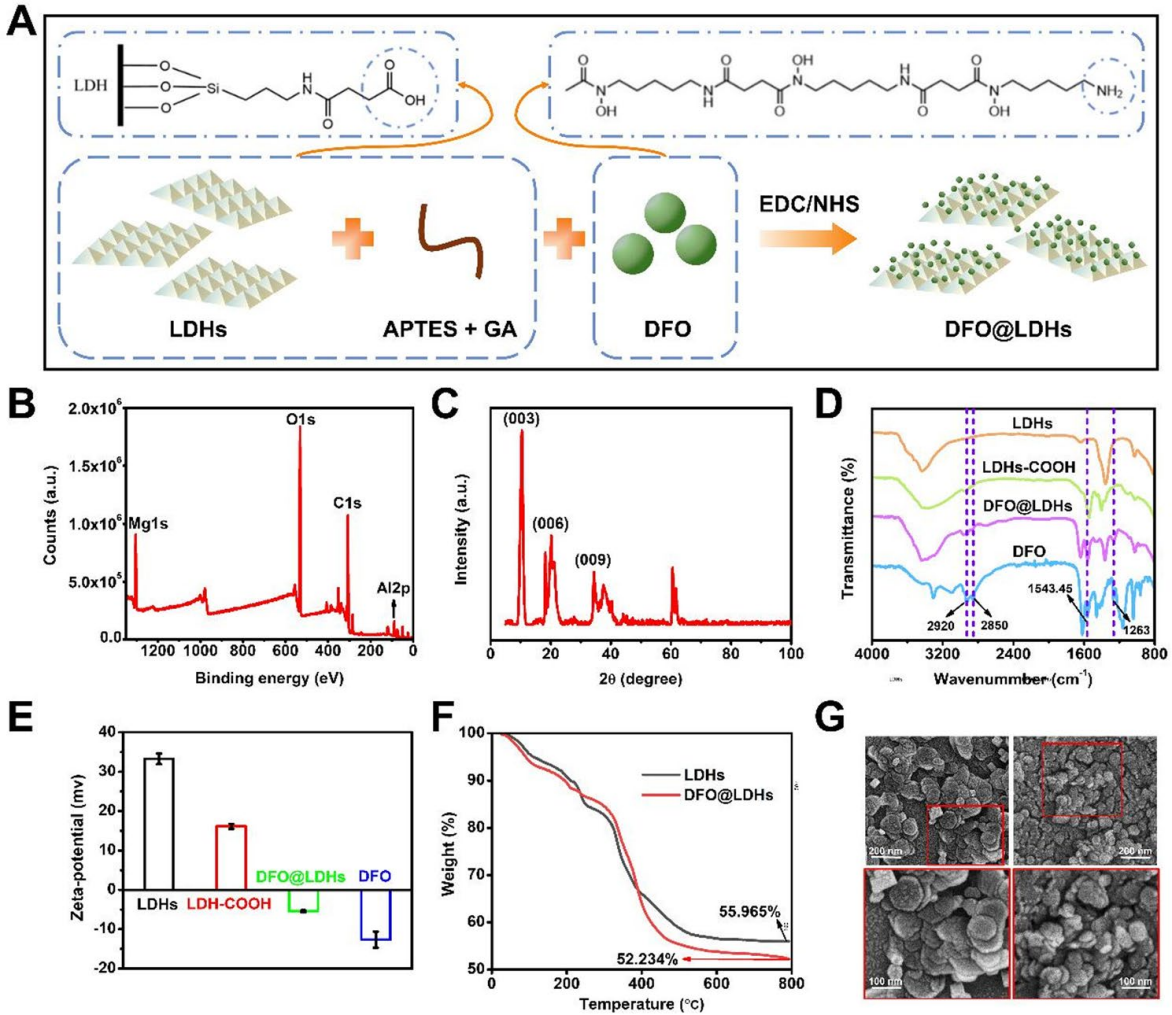
Preparation and characterization of DFO@LDHs. (**A**) Schematic diagram of the synthesis of DFO@LDHs; (**B**) XPS pattern of LDHs; (**C**) XRD pattern of LDHs; (**D**) FT-IR diagrams of LDHs, LDHs-COOH, DFO@LDHs and DFO; (**E**) Zeta potential values of LDHs, LDH-COOH, DFO@LDHs, and DFO; (**F**) Thermogravimetric diagrams of LDHs and DFO@LDHs; (**G**) SEM images of LDHs and DFO@LDHs

The schematic diagram of preparation of DFO@LDHs is shown in Fig. 2A. There are a large number of hydroxyl groups on the surface of LDHs laminates, which can be functionalized by electrostatic interaction, hydrogen bonding and hydroxyl covalent connection. In this study, LDHs were aminated and carboxylated successively by APTES and glutaric anhydride (GA). The amino groups in DFO are equipped to react with carboxylated LDHs to achieve drug grafting. LDHs was successfully prepared by hydrothermal method with Al(NO_3_)_3_·9H_2_O and Mg(NO_3_)_2_·6H_2_O as raw materials (Fig. 2B and C). The prepared LDHs were characterized by X- ray photoelectron spectroscopy (XPS). The XPS data of LDHs showed the characteristic peaks of 1304.8 eV (Mg 1 s), 531.08 eV (O 1 s), 285.08 eV (C 1 s) and 75.08 eV (Al 2p) (Fig. 2B), which were related to the previous ones [27]. In addition, the prepared LDHs were characterized by X-ray diffraction (XRD). LDHs showed characteristic diffraction peaks at 2θ = 10.41° (003), 2θ = 20.21° (006) and 2θ = 34.43° (009) (Fig. 2C) [27].

The grafted LDHs were then characterized. The FT-IR spectra of each sample are shown in Fig. 2D. A broad absorption band appeared near 3433.26 cm^−1^, which was attributed to the hydroxyl layer and interlayer water of LDHs, and the characteristic absorption peak at 1647.62 cm^−1^ was due to the deformation vibration of water molecules. Due to the presence of nitrate ions between the LDHs layers, a sharp and intense band corresponding to NO^3-^ vibration appeared at 1357.09 cm^−1^, indicating the successful preparation of LDHs. Compared with the original LDHs, the peak of LDHs-COOH at 1543.45 cm^−1^ is due to N-H bending vibration, which is the characteristic peak of amide bond; the peaks of LDHs-COOH near 2920 cm^−1^ and 2850 cm^−1^ are the stretching vibration peaks of methylene, all of which indicate that LDHs are successfully carboxylated. In the spectrum of DFO@LDHs, a characteristic peak of DFO appeared at 1262.95 cm^−1^, which may be the N-O bond stretching vibration peak of the -N-OH group in DFO, which further verifies the successful loading of DFO in DFO@LDHs. The average Zeta-potentials of LDHs, LDHs-COOH, DFO@LDHs, and DFO were measured to be 33.267, 16.133, −5.443, and −12.633 mV, respectively (Fig. 2E). After LDHs were carboxylated, the potential of LDHs-COOH decreased because the carboxyl group was negatively charged, indicating that the functionalization grafting of LDHs was successful. The potential of DFO@LDHs exhibited a further reduction, which is attributed to the partial shielding of the cationic sites on the surface of LDHs by DFO molecules, further illustrating the successful grafting of DFO on LDHs. In addition, the thermal stability of LDHs and DFO@LDHs was evaluated by TGA. LDHs will experience three obvious weight loss during the heating process (Fig. 2F). The first stage is when the temperature is 50 ~ 200 ℃, LDHs loses interlayer water after heating, and the interlayer spacing becomes smaller; 200 ~ 400 ℃ is the second stage, and the interlayer water continues to be lost at high temperature. At this stage, carbonate ions begin to decompose, leading to a structural transformation of LDHs into a composite metal oxide. The weight loss observed in the third stage is attributed to the complete decomposition of carbonate ions and the full removal of residual water. At this time, the temperature is as high as 400 ~ 500 ℃, and the structure of LDHs changes to form a strong alkaline metal compound. When heated to 800 ℃, the remaining weight of LDHs and DFO@LDHs accounts for 55.97% and 52.23% of the original mass, respectively. This mass difference is mainly attributed to the pyrolysis of DFO in the temperature range of 50 ~ 500 ℃, which proves the successful grafting of DFO.

The microscopic morphology of LDHs and DFO@LDHs was analyzed using SEM. The prepared LDHs have a typical hexagonal morphology of layered double hydroxides and are about 100 nm in size. DFO@LDHs are stacked together in different directions, and the aggregates are irregular, but the single DFO@LDH still has a sheet structure and is about 50 nm in size (Fig. 2G). The smaller size of a single DFO@LDH may be because LDHs are sensitive to acidic environments. The addition of glutaric anhydride during the carboxylation of LDHs changes the structure of a small part of its surface, but the smaller surface area is more conducive to the release of degradable drugs during the sustained release process.

In order to evaluate the loading efficiency and release behavior of DFO in LDHs, the absorption of Fe^3+^ in solution by DFO was first evaluated. DFO can chelate with Fe^3+^ to form a DFO-Fe^3+^ complex, which causes the color of the solution to change (Fig. S1A). The complex has an obvious characteristic absorption peak at 430 nm (Fig. S1B). Therefore, 430 nm is chosen as the detection wavelength for the determination of DFO content in this experiment. Different concentrations of DFO solutions (0 ~ 1 mg/mL) were reacted with 3mM FeCl_3_.6H_2_O to establish a standard curve (Fig. S1C), and the *EE* (Encapsulation efficiency) and *LE* (Loading efficiency) of DFO drugs in five different DFO@LDHs reaction systems were calculated (Fig. S1D). With the gradual increase of DFO added to the reaction system, both the *EE* and *LE* initially increased, followed by a subsequent decrease. The data showed that the drug loading rate of DFO@LDHs was the highest when 10 mg of DFO was put into the grafting reaction system, which was about 12.43 ± 0.15 wt% (Fig. S1D). Therefore, this concentration of DFO@LDHs was selected for subsequent measurement and characterization.

Based on the Zeta - potential measured in the previous article, it is evident that LDHs carry a positive charge while DFO exhibits electronegativity. In order to prove that DFO is loaded on DFO@LDHs not only through electrostatic adsorption, the remaining DFO content in the supernatant was measured after the reaction in both the adhered (left side of the red box) and grafted (right side of the red box) DFO-LDHs systems, and the drug loading rate was subsequently calculated. The results showed that the drug loading rate of DFO@LDHs in the grafted system (12.43%) was much higher than that of DFO@LDHs in the adhered system (0.95%), which further demonstrated that DFO was successfully grafted on LDHs (Fig. S1E).

The drug release behavior of DFO@LDHs was investigated in vitro, and the release rate of DFO was determined by UV spectrophotometry. The release rate of DFO was the fastest on the first day, with a cumulative release of 29.43%, which may be due to the sudden release of DFO through electrostatic adsorption. The cumulative release of DFO on the third day was 48.034%. In the subsequent release time, the release rate of DFO exhibited a significant slowdown, and the cumulative release percentage on 25 days was 56.42% (Fig. S1F).

### Characterization of D@LG/D@LP biomimetic scaffolds

In order to determine the optimal concentration for loading DFO@LDHs into the scaffold, a series of DFO@LDHs concentrations (2 wt%, 1 wt%, 0.5 wt%, 0.25 wt%, and 0.125 wt%) were selected for loading for the fabrication of D@LG/D@LP-2, D@LG/D@LP-1, D@LG/D@LP-0.5, D@LG/D@LP-0.25, and D@LG/D@LP-0.125 scaffolds, respectively. HUVECs and 3T3-E1 were used for testing the biocompatibility for optimizing the loading concentration of DFO@LDHs. The HUVECs proliferation results showed that the cell growth rate in the extracts of both D@LG/D@LP-0.5 and D@LG/D@LP-0.25 was the fastest and there was no significant difference between the two (Fig. S2A). Moreover, the cell growth rate of 3T3-E1 in the extract of D@LG/D@LP-0.5 was the fastest (Fig. S2B). Therefore, 0.5 wt% was identified as the optimal concentration for loading DFO@LDHs into the scaffold. For convenience, D@LG/D@LP-0.5, which was determined to have the optimal drug loading concentration as described above, is referred to as D@LG/D@LP in the subsequent text. To ensure consistency and control variables across experimental groups, LG/LP also utilized 0.5 wt% as the concentration of LDHs loaded into the scaffold material.

**Fig. 3:**
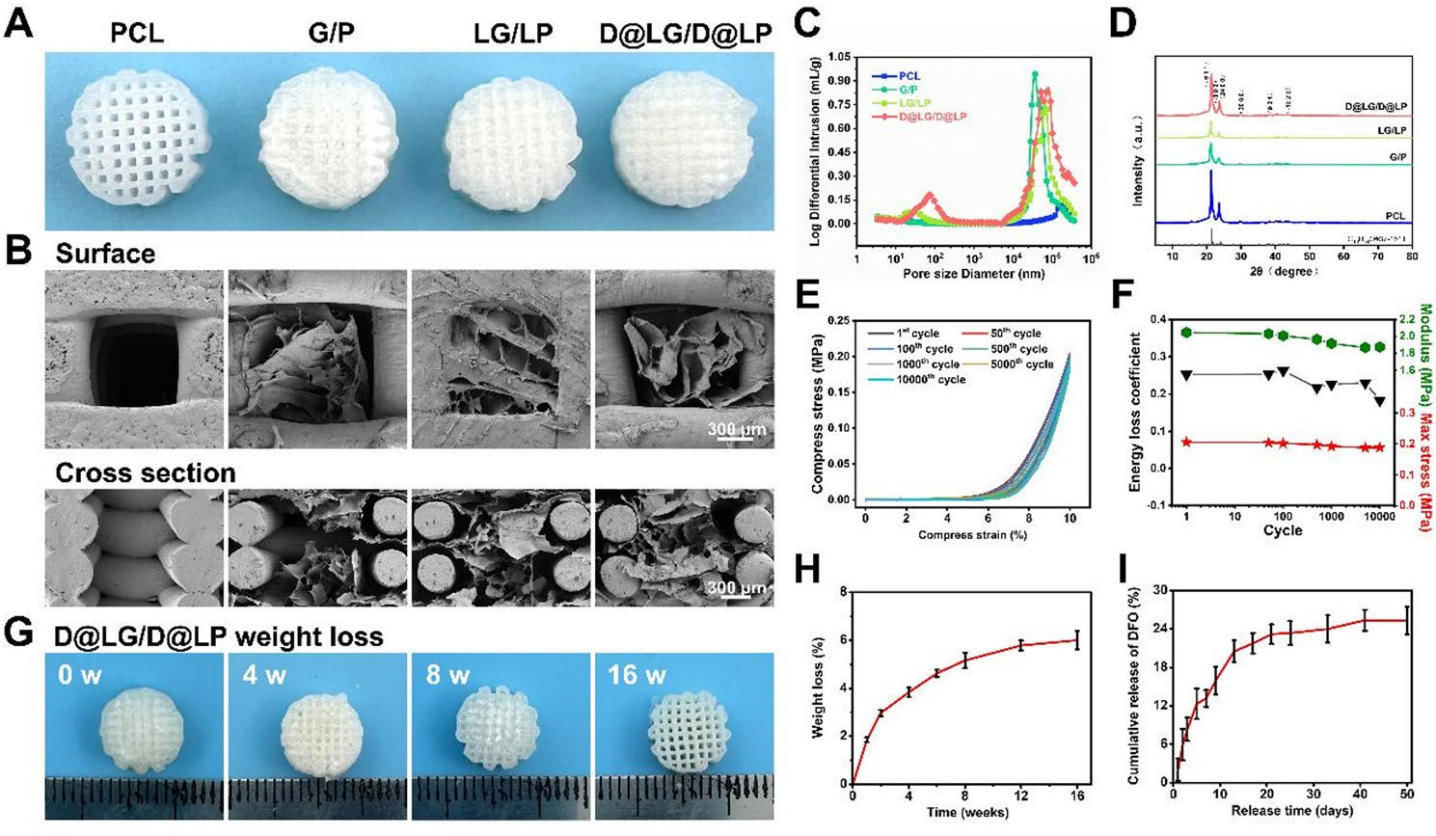
Characterization of scaffolds. (**A**) Representative photos and (**B**) SEM images of PCL, G/P, LG/LP, and D@LG/D@LP; (**C**) The size and density of the pores within different scaffolds; (**D**) X-ray diffraction patterns of different scaffolds; (**E**) Compression curves of D@LG/D@LP scaffold under 10,000 cycles; (**F**) Storage modulus, energy loss coefficient, and max stress of D@LG/D@LP scaffold at 10,000-cycle compression; (**G**) Representative photos of D@LG/D@LP; (**H**) degradation curves of D@LG/D@LP immersed in PBS at different time points; (**I**) DFO release curve of D@LG/D@LP in PBS

 G/P, LG/LP, and D@LG/D@LP were observed in macroscopic morphology to be formed by hydrogel filling within 3D-printed scaffolds (Fig. 3A). In the SEM images of the surface (above) and cross-section (below) of PCL, G/P, LG/LP, and D@LG/D@LP, respectively (Fig. 3B). From the surface image, it is clear that the PCL scaffold has a square hollow structure, while the cavities of the G/P, LG/LP, and D@LG/D@LP are obviously filled with hydrogels; the surface of the PCL scaffold is smooth, while the D@LG/D@LP has a rough cross-section due to the addition of DFO@LDHs. In the cross-section, the hydrogel in the composite scaffold presents an obvious porous structure, which is conducive to cell growth. In addition, it can be clearly observed from the cross-section of the D@LG/D@LP scaffold that the hydrogel surface is rougher, because the DFO@LDHs are evenly dispersed and embedded in the entire hydrogel matrix. Subsequently, we characterized the pore size and distribution within the hydrogels using mercury intrusion porosimetry. In G/P, LG/LP, and D@LG/D@LP hydrogels, pore sizes predominantly ranged from 20 to 250 μm. Notably, D@LG/D@LP exhibited an additional peak at 30 to 100 nm (Fig. 3C, Table S1). As the hydrogel undergoes dynamic degradation, the evolving pore structure enables specific responses that effectively modulate diverse cellular behaviors. XRD was used to characterize the crystalline structure of D@LG/D@LP, including the states before and after loading LDH nanosheets and HA-GMA hydrogel into PCL scaffold (Fig. 3D). The XRD patterns revealed that the composite scaffolds retained all major diffraction peaks of the pure PCL scaffold, indicating that the fundamental crystalline phase of PCL, serving as the printed scaffold, was fully preserved after composite formation. However, detailed comparison revealed subtle structural alterations induced by material composite formation. The most pronounced change occurred in the diffraction peak corresponding to the (921) crystal plane. This peak position shifted significantly from 38.40 to 36.40°, while the associated d-spacing increased from 2.35 to 2.47 Å. This pronounced shift indicates that during PCL crystallization, the incorporated nanosheets likely functioned as heterogeneous nucleation sites. They exerted significant steric hindrance effects on molecular chain stacking along specific crystal orientations, leading to lattice distortion or expansion in those directions. Such interactions favor enhanced interfacial bonding strength between the PCL matrix and nanosheets. Concurrently, we observed only a negligible shift in PCL’s main peak (−611) after composite formation (21.44° vs. 21.38°). This confirms the overall structural stability of the PCL framework while suggesting that the introduction of nanosheets and HA-GMA hydrogel exerts a minor thermodynamic influence on PCL crystallization, potentially slightly altering its crystallinity or microcrystal size (Table. S2). XRD results confirm the successful construction of a multilevel composite scaffold featuring a stable PCL crystalline framework. The incorporation of nanosheets not only preserved the printability of PCL but also enabled robust integration from the microscopic crystalline structure to the macroscopic scaffold through molecular-level interactions with PCL. This synergistic interaction among multiple components holds promise for significantly enhancing the mechanical properties and biological functionality of the composite scaffold.

In addition, this study evaluated the hydrophilicity of PCL, HA/GMA, G/P, and D@LG/D@LP by measuring water contact angles (Fig. S3). The results showed that PCL had poor hydrophilicity, HA/GMA scaffolds had excellent hydrophilicity, and composite scaffolds G/P, LG/LP, and D@LG/D@LP had great hydrophilicity, which was largely attributed to the introduction of HA/GMA, a material with excellent hydrophilicity. The scaffold D@LG/D@LP, boasting outstanding hydrophilicity, has the capacity to effectively facilitate cell adhesion and proliferation, and significantly promote cell osteogenic differentiation.

As a bone defect implantation scaffold, it must not only provide mechanical support but also withstand cyclic physiological loads [28]. We conducted cyclic 10% deformation compression tests on PCL and D@LG/D@LP scaffolds to investigate their mechanical properties and stability. Although PCL scaffolds exhibit high compressive strength, their compressive capacity significantly diminishes with increasing cycle numbers (Fig. S4). Although the mechanical properties of D@LG/D@LP scaffolds were reduced compared to PCL scaffolds, they remained within the compressive strength range of cancellous bone (0.1–16 MPa) [29]. Moreover, even after 10,000 compression cycles, they maintained stable mechanical responsiveness without noticeable stress decay or abnormal strain changes (Fig. 3E). Furthermore, the storage modulus, maximum stress, and energy loss coefficient remained stable with increasing compression cycles (Fig. 3F), indicating excellent fatigue resistance. The faster recovery rate and superior fatigue resistance suggest that D@LG/D@LP scaffolds are highly suitable as bone tissue scaffolds under physiological loading conditions, capable of withstanding cyclic mechanical stresses.

PCL exhibits a slow degradation rate, typically requiring more than a year to undergo significant degradation. Studies have shown that 3D-printed PCL is able to retain its compressive strength at a consistent level even after 6 months of degradation in PBS [30, 31]. Therefore, the PCL scaffold incorporated with DFO@LDHs has the ability to provide long-term mechanical support after implantation, meeting both the temporal and mechanical requirements for bone defect reconstruction. The D@LG/D@LP was immersed in PBS solution and placed in a 37 ℃ shaker to observe the in vitro degradation of the scaffold. Freeze-dried samples were collected after 4, 8, 12, and 16 weeks. From the photo (Fig. 3G), the D@LG hydrogel filled in the scaffold gap degraded rapidly. In addition, the mass loss curve of the D@LG/D@LP was recorded. The percentage of weight loss gradually increased with the degradation time. After 16 weeks of degradation, the D@LG hydrogel was basically degraded, and the weight loss percentage of the D@LG/D@LP was determined to be about 6.01% (Fig. 3H). The D@LG hydrogel emulates the natural microenvironment, thereby facilitating the ingrowth of blood vessels. Meanwhile, the swift degradation of this material holds the potential to establish sufficient space for the ingrowth of bone tissue.

The drug release behavior of D@LG/D@LP was evaluated in vitro using the same method as described in Sect. “Physicochemical properties of DFO@LDHs nanoparticles and DFO release properties”, which was employed to determine the release rate of DFO from DFO@LDHs. The release rate of DFO was very fast in the first 5 days, with a cumulative release of 12.34 ± 2.38%. The release rate of DFO gradually slowed down from 5 to 21 days, with a cumulative release percentage of 23.22 ± 1.52% on the 21 st day. In the subsequent release period, the release rate of DFO slowed down significantly, with a cumulative release percentage of 25.29 ± 2.17% within 50 days (Fig. 3I). Compared with the release rate of DFO in DFO@LDHs, the release rate of DFO in the scaffold slowed down significantly, without sudden release and achieving long-term release. The reason for this drug was controlled release pattern may be related to the different degradation rates of different components in the scaffold, with D@LG degrading faster and D@LP degrading slower. Since bone reconstruction requires rapid angiogenesis to provide adequate nutrition, DFO in the scaffold is released relatively quickly in the early stage and continuously in the later stage, which is conducive to the early stimulation of new blood vessel formation and the subsequent formation of strong bones. It is worth noting the rationale behind the release duration relative to the repair timeline. The sustained release of DFO for approximately 50 days covers the critical window of angiogenesis during the early stages of bone healing (inflammatory and soft callus phases). Since vascularization is a prerequisite for osteogenesis, the early establishment of a vascular network driven by DFO provides the necessary metabolic microenvironment for subsequent bone mineralization. In the later stages (up to 12 weeks), biological requirements shift from neovascularization to matrix maturation and load-bearing, which are supported by the slowly degrading PCL framework and the bioactive ions (Mg^2+^), rather than requiring prolonged DFO stimulation.

Regarding the metabolic fate of the inorganic components, LDHs exhibit a distinct biodegradation profile compared to non-degradable nanoparticles. Due to their acid-sensitivity, LDHs undergo rapid dissolution within the acidic microenvironment of lysosomes (pH ≈ 4.5–5.0) following cellular endocytosis [32, 33]. This process decomposes the nanosheets into biocompatible magnesium (Mg^2+^) and aluminum (Al^3+^) ions. The released Mg^2+^ contributes to bone metabolism, as ditscussed earlier, while the trace amounts of Al^3+^ and solubilized ligands enter the systemic circulation and are primarily eliminated through renal clearance, thus avoiding long-term accumulation in the reticuloendothelial system (RES) or significant systemic toxicity [34].

It should be noted that the scaffolds underwent sterilization via 75% ethanol fumigation and UV irradiation prior to application. Although DFO is a bioactive peptide derivative sensitive to oxidation, its intercalation within the LDH nanosheets provides a shielding effect against UV degradation. Furthermore, while ethanol induces temporary dehydration of the HA-GMA hydrogel, the crosslinking network remains intact, allowing the hydrogel to fully recover its swollen porous structure upon rehydration in physiological fluids. The robust biological activity observed in subsequent experiments confirms that this sterilization protocol preserves the structural and functional integrity of the composite scaffold.

### Biocompatibility and angiogenic activity of D@LG/D@LP biomimetic scaffolds in vitro

**Fig. 4:**
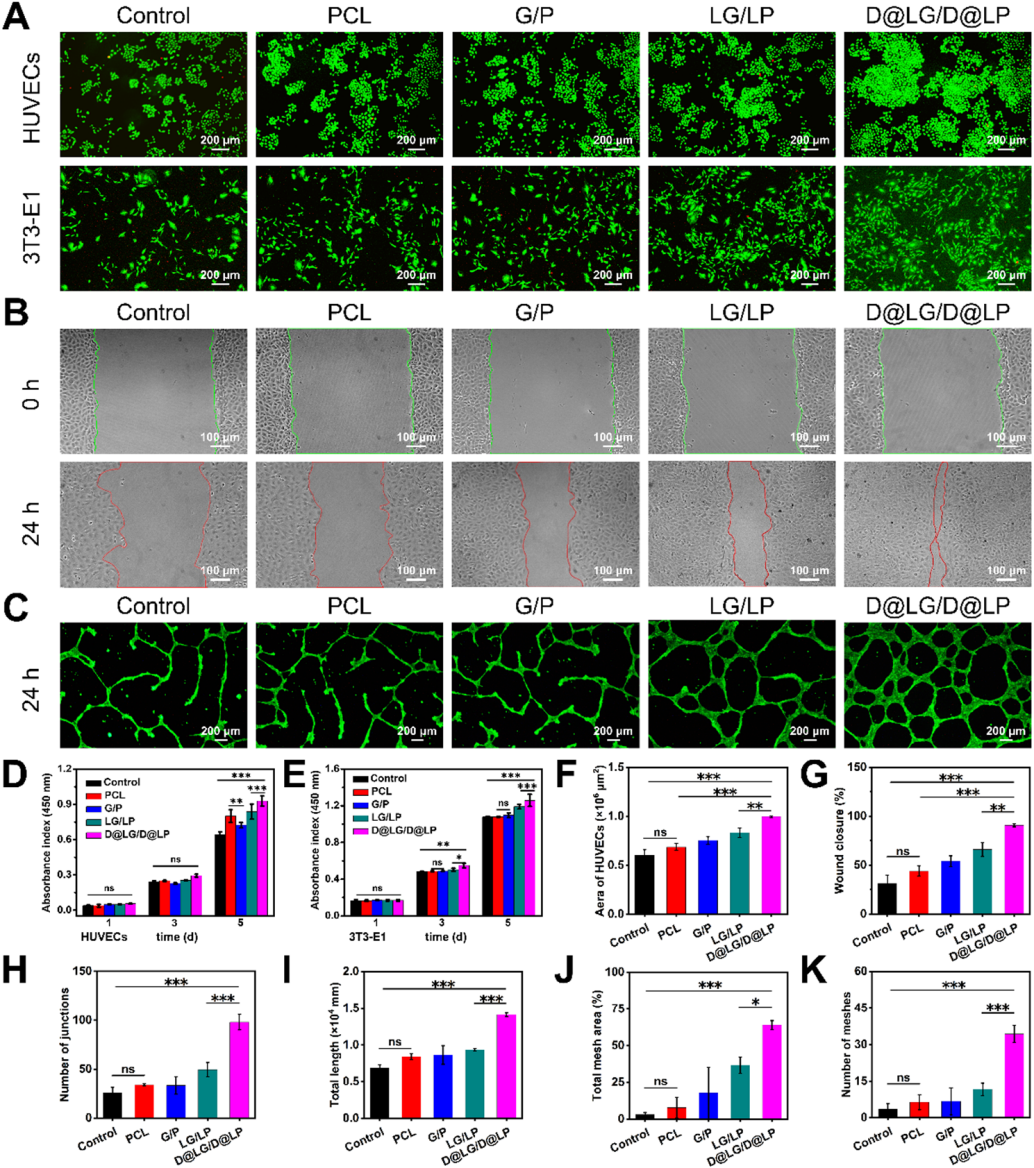
Effect of scaffolds on cell proliferation, migration and angiogenesis. (**A**) Live-dead staining of HUVECs and 3T3-E1cultured in the leaching solutions of PCL, G/P, LG/LP, and D@LG/D@LP; (**B**) Scratch assay images of HUVECs cultured in the leaching solutions of different scaffolds before and after 24 h; (**C**) Calcein-AM staining images of lumen formation in HUVECs under a fluorescence microscope after 24 h; (**D**) Quantitative analysis of the proliferation of HUVECs in the leaching solutions of different scaffolds; (**E**) Quantitative analysis of the proliferation of 3T3-E1 cells in the leaching solutions of different scaffolds. (*n* = 4; ^*^p < 0.05, ^**^p < 0.01, ^***^p < 0.001, and “ns” indicated no significant difference) (**F**) Quantitative results of the scratch healing area; (**G**) Quantitative results of the scratch healing percentage; (**H-K**) Quantitative evaluation of angiogenesis parameters, including the number of connections, total length, number of meshes, and total mesh area percentage. (*n* = 3; *p < 0.05, **p < 0.01, and ***p < 0.001, and “ns” indicated no significant difference)

To evaluate the biocompatibility of PCL, G/P, LG/LP, and D@LG/D@LP, HUVECs and 3T3-E1 cells were cultured in the extracts of these scaffolds for 1, 3, and 5 days, followed by assessment using CCK-8 assay and live-dead cell staining (Fig. 4A, D and E). After 1, 3, and 5 days, HUVECs and 3T3-E1 showed vigorous proliferation ability in all groups; the D@LG/D@LP group exhibited the highest proliferation rates for both HUVECs and 3T3-E1 cells, likely attributed to the concentration of DFO released from the scaffold in the leaching solution, which promotes cell proliferation.

Generally, angiogenesis is initiated from pre-existing vasculature by sprouting endothelial cells (ECs) [35]. The physiological process of angiogenesis includes multiple stages. The endothelial matrix is first degraded, and ECs migrate and proliferate to form a tube with a lumen. Finally, blood flow begins [36, 37]. Therefore, in order to simulate the in vivo angiogenesis process, the endothelial cell scratch test and tubule formation test have become classic in vitro methods for evaluating angiogenic potential [38].

The optical microscope image of the cell scratch test shows obvious signs of cell migration in the corresponding images of the four scaffold groups (Fig. 4B). Interestingly, the D@LG/D@LP group demonstrated the highest level of cell migration and exhibited superior scratch healing performance, with a scratch healing area of 1.00 ± 0.01% mm² (Fig. 4F) and a scratch healing rate of 90.69 ± 1.43% (Fig. 4G).

Matrigel reflects the ability of endothelial cells to remodel the extracellular matrix (ECM) [39]. In order to simulate angiogenesis in a 3D environment, a tubule formation assay was conducted. After 24 h of incubation, different degrees of tubular structure formation were observed on Matrigel. Fluorescence imaging results showed that more tubular structures were formed in the D@LG/D@LP group (Fig. 4C). More notably, a network more similar to capillaries could be observed in the D@LG/D@LP group. In addition, there were significant differences in the number of junctions, total length, number of meshes, and percentage of total mesh area among the four experimental groups (Fig. 4H and K). The tubular formation ability of the LG/LP group was significantly higher than that of the PCL and G/P groups. This phenomenon may be attributed to the free Mg²⁺ released from LDHs. These liberated ions have the capacity to boost the secretion of VEGF. This, in turn, induces the expression of HIF-1α and VEGF-A, thereby promoting vascularization [40, 41]. In contrast, the calculated parameters of the D@LG/D@LP group increased sharply, and the number of junctions and total length were significantly higher than those of the other groups. The above results indicate that the D@LG/D@LP is in a position to release a sufficient dose of DFO to regulate the biological functions of HUVECs. These functions encompass guiding cell migration and promoting the formation of tubular structures. In conclusion, D@LG/D@LP have significant pro-angiogenic potential.

### Osteogenic activities of scaffolds in vitro

**Fig. 5:**
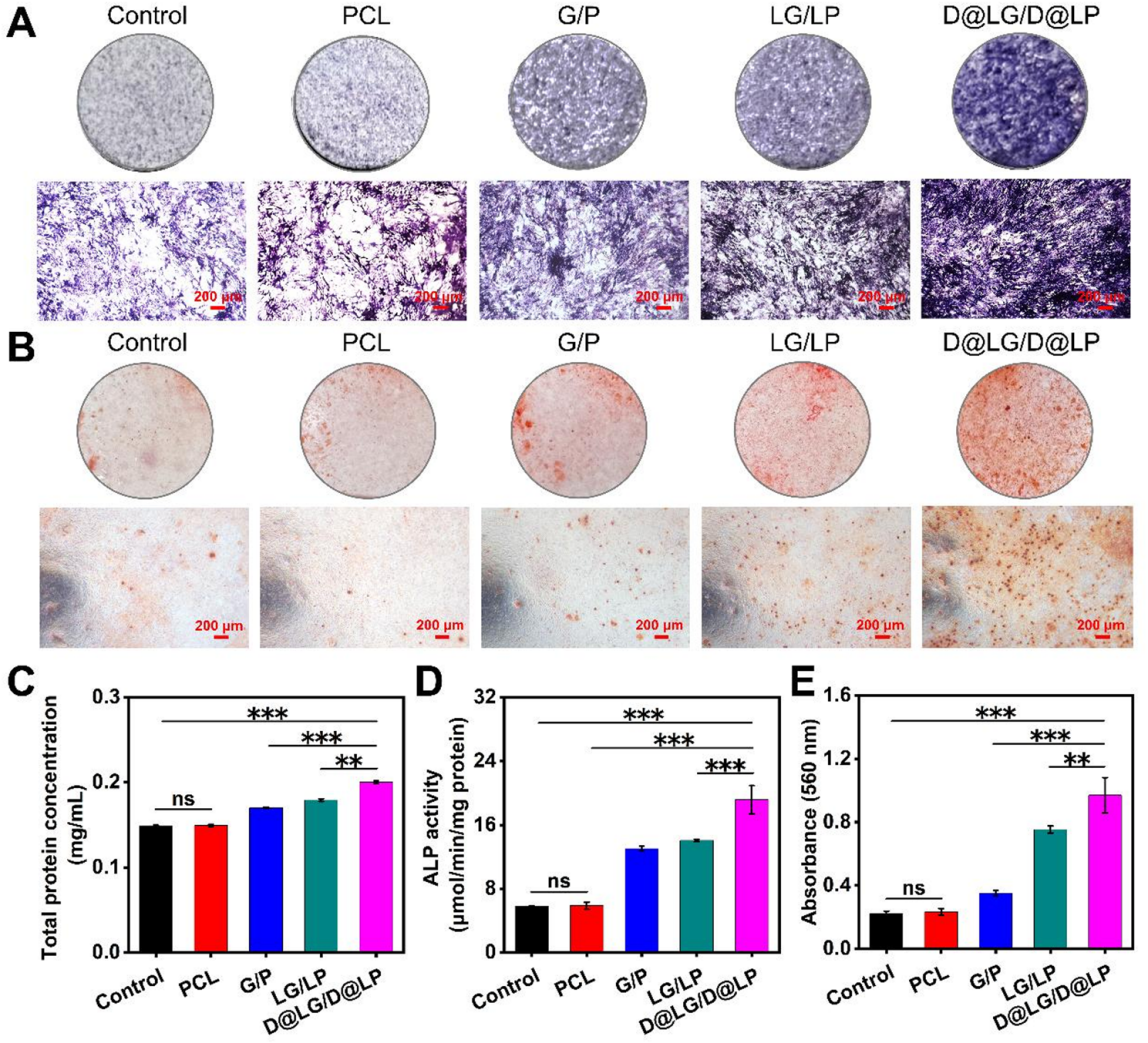
In vitro osteogenic potential of scaffolds. (**A**) ALP staining images of 3T3-E1 in different scaffold extracts on day 14; (**B**) ARS staining images of 3T3-E1 in different scaffold extracts on day 21; (**C**) Total protein concentration of quantified cells in ALP quantitative analysis on day 14; (**D**) Quantitative results of ALP activity on day 14; (**E**) Quantitative results of ARS on day 21. (*n* = 4; *p < 0.05, **p < 0.01, and ***p < 0.001, and “ns” indicated no significant difference)

Osteogenic differentiation represents a critical process in bone regeneration, and its activity is assessed using a range of assay methods. This study carefully evaluated the impact of these scaffolds on 3T3-E1 osteogenic differentiation through quantitative and qualitative analysis using ALP and ARS staining [42]. First, to evaluate the osteogenic differentiation potential of these scaffolds, ALP staining and activity assay were performed on 3T3-E1 after 14 days of culture. It can be observed that the D@LG/D@LP group has the strongest ALP staining in the images corresponding to the four groups of scaffolds, followed by LG/LP, G/P, PCL, and the control group (Fig. 5A). Quantitative analysis of activity after 14 days showed consistent trends in results (Fig. 5D). The above results confirmed that the D@LG/D@LP had outstanding osteogenic potential. Alizarin red staining was used to determine the degree of mineralization of ECM in 3T3-E1 cultures, reflecting the later osteogenic activity. After 21 days of osteogenic induction, ARS staining was conducted on 3T3-E1 cells (Fig. 5B). The light microscope images revealed positive ARS staining results in all groups, with the D@LG/D@LP group exhibiting a significant increase in mineralized nodule deposition. In contrast, no significant difference was observed between the PCL and control groups. These results were also confirmed by quantitative analysis of ECM mineralization (Fig. 5E). Cells incubated with D@LG/D@LP leach solution had the most calcium deposition and the highest degree of mineralization, so the osteogenic activity of the D@LG/D@LP in the later stage is also very superb. The excellent osteogenic potential of the D@LG/D@LP for 3T3-E1 may be mainly attributed to the synergistic effect of Mg^2+^ and DFO. As an essential ion involved in numerous physiological processes, Mg²⁺ enhances osteoblast activity and ALP activity while promoting the production of type I collagen in the extracellular matrix and the maturation of extracellular matrix minerals, thereby facilitating bone repair [43–45]. DFO effectively enhances tissue vascularization, thereby promoting bone regeneration [46]. In summary, the D@LG/D@LP has excellent osteogenic induction ability, which is mainly attributed to the free Mg^2+^ in DFO@LDHs and the sustained release of DFO as well as the synergistic effect of their combined action.

### In vivo bone regeneration assessment

**Fig. 6:**
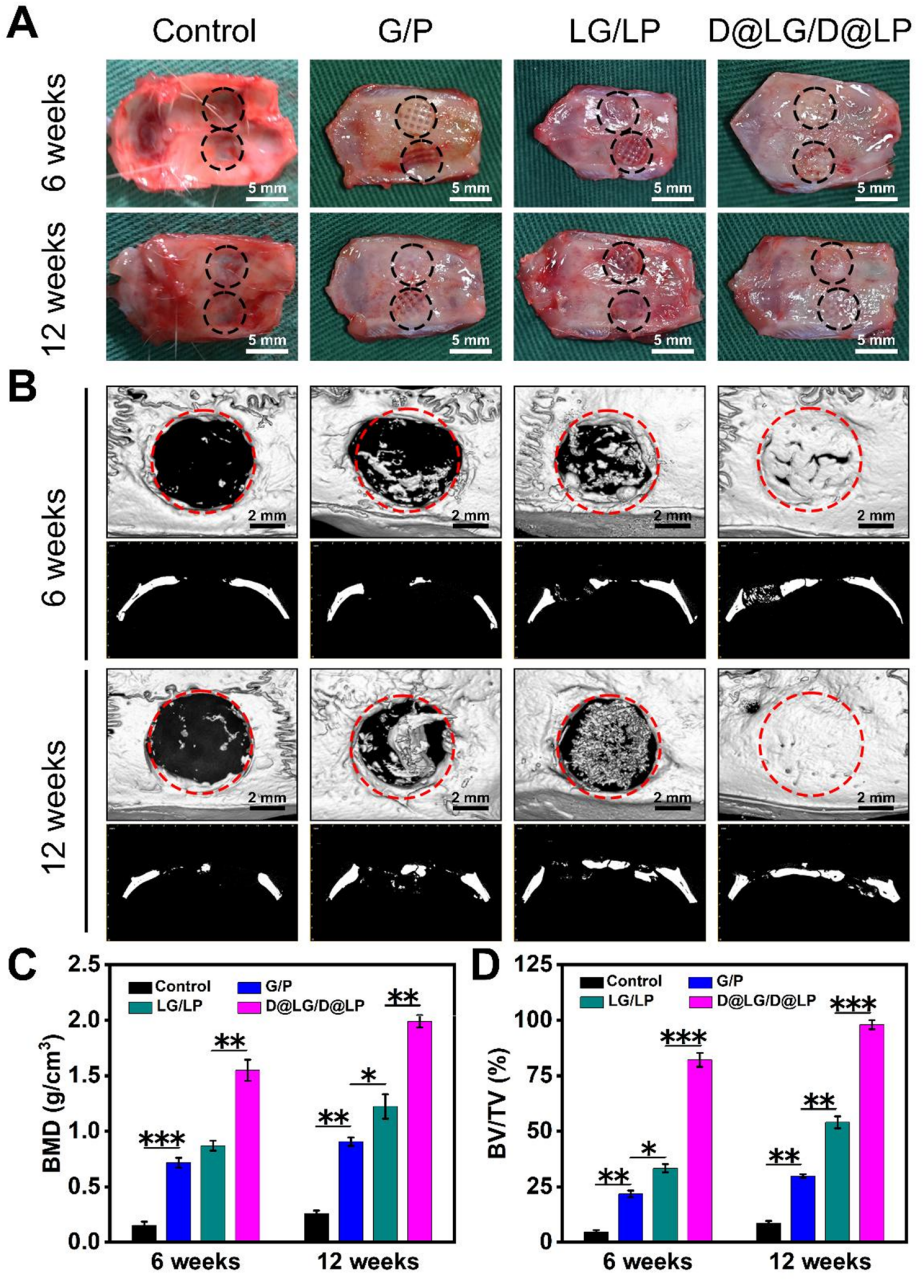
Bone regeneration in a 5 mm diameter rat skull defect model. (**A**) Representative images of rat cranial defect models at 6- and 12-weeks post-implantation. The black circle indicates the bone defect area and the implanted scaffold; (**B**) Representative micro-CT images at 6- and 12-weeks post-implantation, including cross-sectional and coronal views, with the red circle highlighting the bone defect area; (**C**) BV/TV and (**D**) BMD calculated from the micro-CT image results. (*n* = 5; *p < 0.05, **p < 0.01, and ***p < 0.001, and “ns” indicated no significant difference)

To evaluate the osteogenic potential of D@LG/D@LP, a rat cranial defect model with a diameter of 5 mm was established in this study to assess the bone regeneration capability of the scaffold (Fig. 6A). To ensure the consistency of the animal model, macroscopic observation and micro-CT scanning were performed immediately after surgery (Day 0). Uniform critical-sized defects were successfully created across all groups, providing a consistent baseline for subsequent evaluation (Fig. S5). Since the cell experiment in the previous part of this paper has confirmed that there is no significant difference between the PCL group and the control group, the animal experiment in the later part of this paper only set up a control group and three experimental groups of G/P, LG/LP, and D@LG/D@LP. Different scaffolds were implanted in rats for 6 weeks and 12 weeks, respectively, followed by analysis of bone regeneration performance using micro-CT, histological staining, and immunofluorescence staining [47, 48].

Micro-CT examinations were performed at 6 weeks and 12 weeks, and reconstructed micro-CT images of the new bone were captured (Fig. 6B). After 6 weeks, the control group and the G/P group exhibited minimal new bone formation and larger defect areas, while the LG/LP and D@LG/D@LP groups demonstrated limited new bone formation at the defect site. Compared with the G/P and LG/LP groups, the D@LG/D@LP group had the largest new bone coverage area. After 12 weeks of implantation, the defect area of most scaffold groups was reduced, and except for the G/P group, the other groups had a large area of bone defects covered by new bone. It is worth noting that the defect area of the D@LG/D@LP group was significantly shrunk, the new bone at the edge of the defect was fused in large quantities, and the amount of new bone formation was significantly higher than that of the control group and the G/P and LG/LP groups. Quantitative morphological analysis showed that the BMD of the D@LG/D@LP group was the highest (Fig. 6C), followed by the LG/LP group, the G/P group and the control group. In addition, the trend of the BV/TV ratio was similar (Fig. 6D). The BMD of the D@LG/D@LP group reached 1.99 ± 0.05 g/cm^3^ at week 12, and the BV/TV reached 98.03 ± 1.67%, both of which were significantly higher than those of the LG/LP group (BMD, 1.22 ± 0.09 g/cm^3^; BV/TV, 54.03 ± 2.17%) and the G/P group (BMD, 0.91 ± 0.031 g/cm^3^; BV/TV, 54.03 ± 2.17%). These results indicate that the D@LG/D@LP group is equipped to promote the formation of new bone in vivo.

**Fig. 7:**
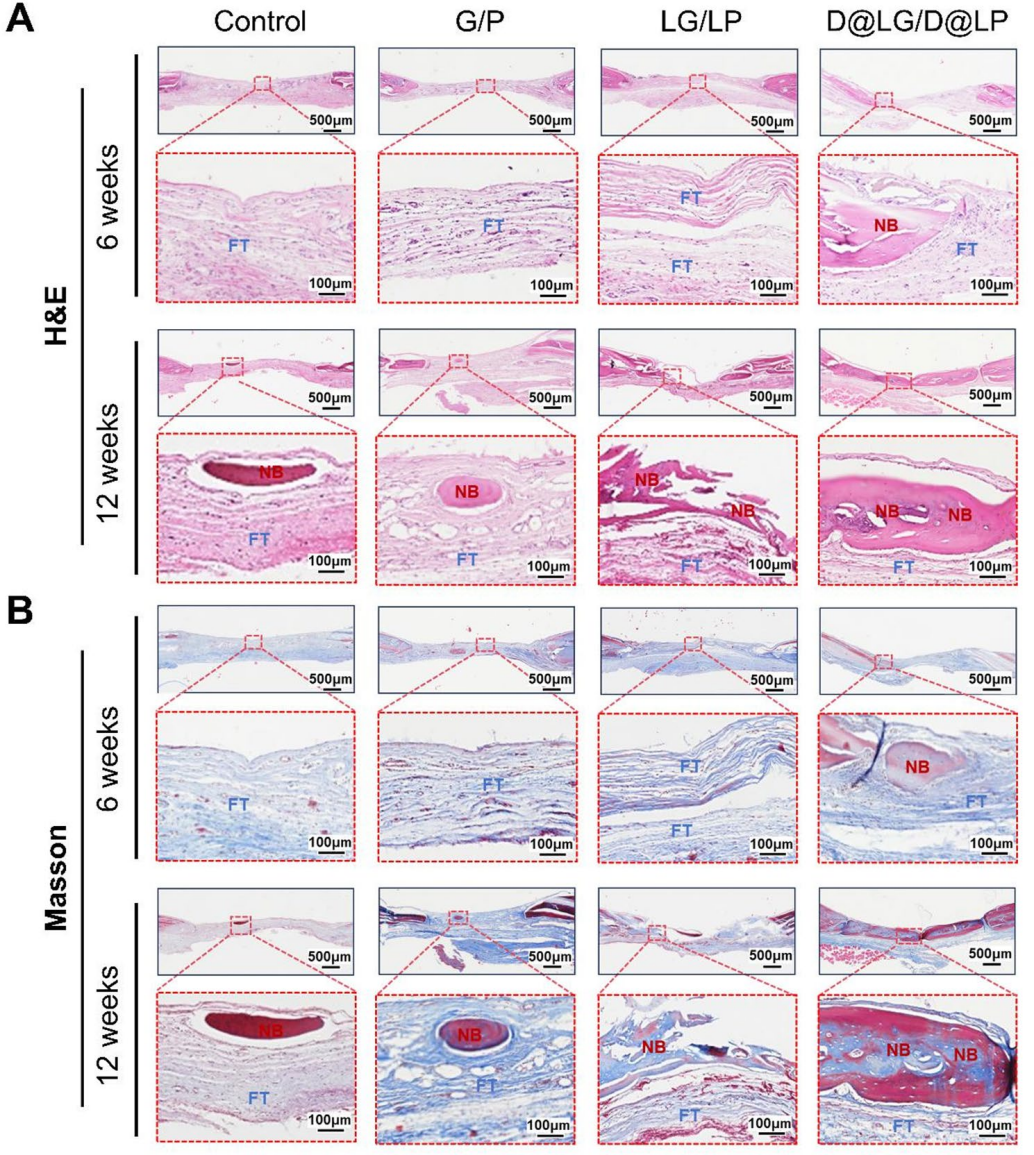
Histological analysis of skull sections at 6 and 12 weeks. (**A**) H&E staining images; (**B**) Masson staining images. The red boxes are enlarged images of the corresponding parts; NB: new bone; FT: fibrous tissue

The formation of new bone at the defect site was further confirmed through hematoxylin and eosin (H&E) staining and Masson’s trichrome staining (Fig. 7). From the H&E images, it is evident that a significant amount of fibrous tissue bridged the original bone edges in the control group at both 6 and 12 weeks (Fig. 7A). After treatment with different scaffolds, new bone formation was observed in the defect area at 12 weeks. Compared to the control group, the G/P, LG/LP, and D@LG/D@LP groups exhibited significantly more new bone formation, with the LG/LP and D@LG/D@LP groups demonstrating superior osteogenic capability. However, the D@LG/D@LP group exhibited the most abundant new bone tissue, with its bone mineralization closely resembling that of the surrounding natural bone tissue. Notably, the central and peripheral regions of the D@LG/D@LP scaffold were nearly completely filled with new bone tissue, indicating that the sustained release of DFO and the free metal ions from DFO@LDHs synergistically enhanced osteogenic differentiation and mineralization within the scaffold, thereby promoting bone defect repair. These data are consistent with the results of micro-CT. Masson trichrome staining revealed that at 6 and 12 weeks, the defect areas of the Control, G/P and LG/LP groups were filled with a large amount of collagen fibers, fibrous tissue and scattered immature bone tissue (blue) (Fig. 7B). The new bone (red) in the D@LG/D@LP group was significantly more mature than that in the LG/LP group. The observations confirmed that bone defect repair in the LG/LP and D@LG/D@LP groups was significantly enhanced and accelerated compared to the other groups, with the D@LG/D@LP group demonstrating superior bone defect repair properties. These findings further indicate that the sustained release of DFO and metal ions synergistically promoted osteogenic differentiation and mineralization within the scaffold, aligning with the expected results.

**Fig. 8:**
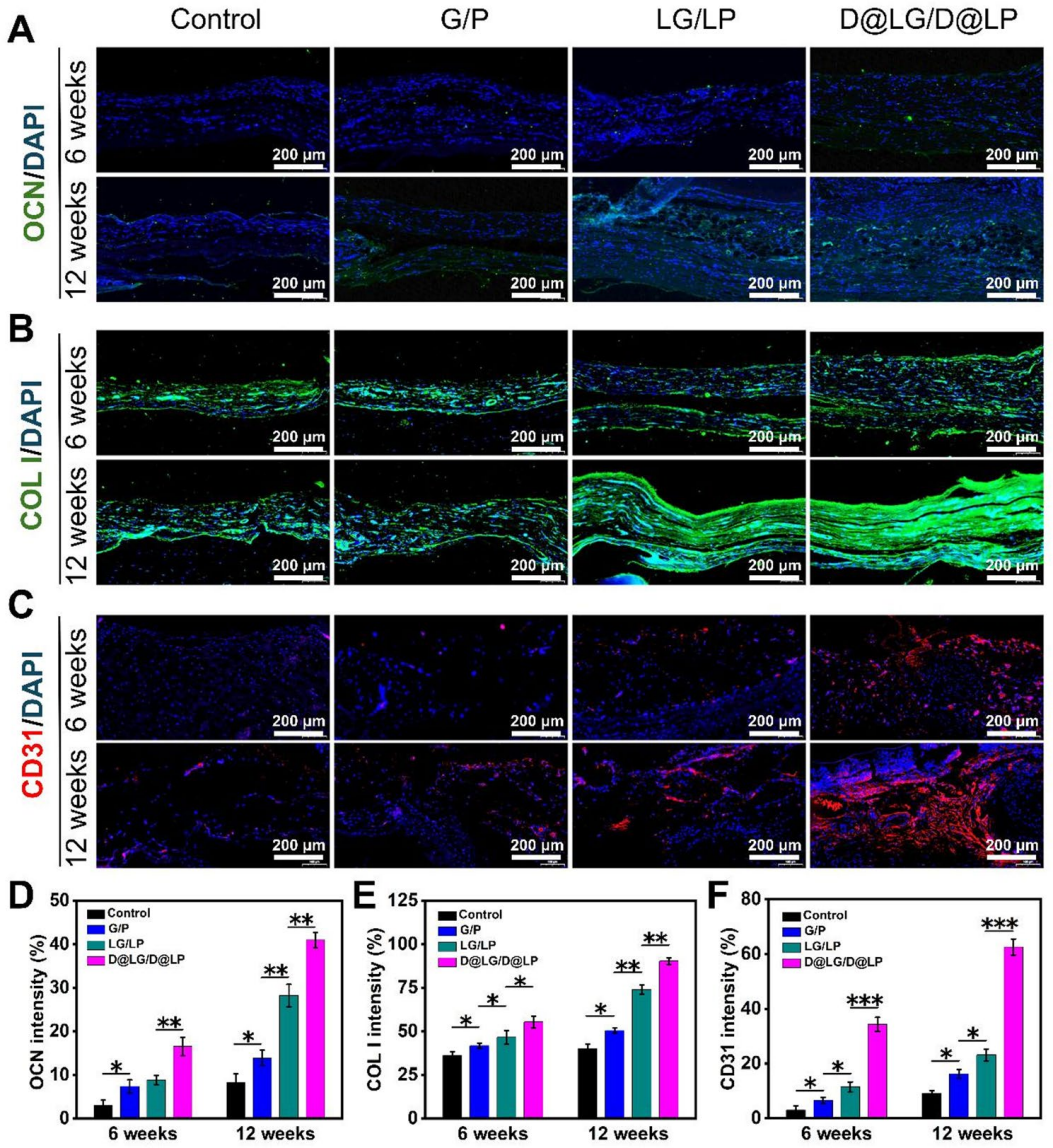
Immunofluorescence staining of decalcified bone sections at 6 and 12 weeks after implantation, with cell nuclei stained with DAPI (blue). (**A**) Fluorescence image of OCN (green); (**B**) Fluorescence image of COL I (green); (**C**) Fluorescence image of CD31 (red); (**D-F**) Quantitative analysis of the positive areas of OCN, COL I, and CD31 expression. (*n* = 5; *p < 0.05, **p < 0.01, and ***p < 0.001, and “ns” indicated no significant difference)

Finally, immunofluorescence staining was performed to validate the angiogenesis-osteogenesis coupling effect following scaffolds implantation in vivo, and the bone regeneration process was assessed by detecting markers associated with angiogenesis and osteogenesis [49]. The expression of osteogenic markers (OCN and COL I) detected by immunofluorescence staining is shown in Fig. 8A and B. The expression of osteogenic markers was minimal in the Control and G/P groups, with significantly weaker signal intensity compared to the LG/LP and D@LG/D@LP groups. The D@LG/D@LP group showed the strongest fluorescence signal, indicating that more bone tissue was formed, which is consistent with the results of micro-CT analysis and histological staining. CD31 is an important marker for angiogenesis [50]. For angiogenesis evaluation, the expression of CD31 was examined. The results revealed positive CD31 staining in the sections of all groups (Fig. 8C). Compared with the other groups, the repair site in the D@LG/D@LP group exhibited abundant CD31 expression. In contrast, the Control and G/P groups displayed minimal new blood vessel formation, while the LG/LP group showed limited angiogenesis. Quantitative analysis of immunofluorescence intensity further confirmed that the expression of OCN, COL I, and CD31 in the D@LG/D@LP group was significantly increased compared with the other groups (Fig. 8D-F), demonstrating its superior performance in inducing both osteogenesis and angiogenesis. The D@LG/D@LP scaffold demonstrated excellent biocompatibility, enhanced osteogenic potential, and robust angiogenic capability, collectively indicating its ability to promote vascularized bone regeneration.

### Clinical translation potential and challenges

Although the rat cranial defect model effectively validated the angiogenic and osteogenic coupling effects of the D@LG/D@LP scaffold, we recognize that clinical bone repair often involves load-bearing long bones (e.g., tibia or femur), which heal via endochondral ossification and are subject to complex mechanical forces. Our mechanical characterization indicated that the PCL framework possesses compressive fatigue resistance comparable to cancellous bone (Fig. 3E-F), suggesting its potential to prevent soft tissue collapse and support early mobilization in load-bearing scenarios. However, future studies utilizing large animal segmental defect models are necessary to verify the scaffold’s stability under dynamic torsion and shear stresses.

Furthermore, translating this “dual-phase” scaffold from bench to bedside presents specific challenges. First, regarding sterilization, while the ethanol/UV protocol used in this study preserved bioactivity, clinical products typically require terminal sterilization (e.g., Gamma irradiation or Ethylene Oxide). Future work must verify the stability of the DFO peptide bonds and HA-GMA crosslinking density under these industrial sterilization conditions. Second, regarding scalability, while 3D printing enables personalized customization for irregular defects, developing an automated, sterile process for uniform hydrogel infusion into the lattice structure will be critical for mass production and quality control.

## Conclusion

In summary, we have successfully fabricated a biomimetic D@LG/D@LP scaffold designed for bone repair. The core innovation of this scaffold lies in its precisely engineered nano-bio interface. This interface, composed of DFO-loaded LDH nanosheets strategically integrated into both the PCL framework and HA-GMA hydrogel, enables spatiotemporally controlled release of bioactive factors. This sophisticated design yields a multiscale porous structure that effectively accommodates the varying sensitivities of host cells to scaffold architecture. More importantly, the nano-bio interface actively orchestrates a cascade of biological events: it preferentially guides endothelial cell infiltration and microvascular network formation within the hydrogel pores, establishing a critical metabolic and microenvironmental foundation. Subsequently, as the scaffold components gradually degrade, the sustained release of DFO and LDHs from this interface persistently stimulates osteogenic activity, leading to sequential and synergistic angiogenesis and bone matrix deposition. This work thus demonstrates a nano-interface-driven strategy for achieving spatiotemporally programmed bone regeneration, offering a promising solution for repairing large bone defects.

## Supplementary Information

Supplementary Material 1.

## Data Availability

No datasets were generated or analysed during the current study.
