# Supplementary material for: Honeycomb-inspired porous biomimetic scaffold with specific adaptability to host cells behavior for bone repair
Source: J Nanobiotechnology. 2026 Feb 13;24:321. doi: 10.1186/s12951-026-04133-7 (PMC13059228; doi:10.1186/s12951-026-04133-7)
Supplement: Supplementary file 1 — Supplementary Material 1. [file 12951_2026_4133_MOESM1_ESM.docx]

Supporting Information

**Honeycomb-inspired porous** **biomimetic scaffold with specific adaptability to host cells behavior for bone repair**

*Langjie Chai ^b,c,1^, Danchi Liu ^d,1^, Jie Chen ^a,1^, Shilin Jiang ^a^, Ye Lu ^a^, Lei Yu ^b,c^, Lu Zhang ^b,c^,Tonghe Zhu ^d^, Chao Liu ^f^, Chenglin Yang ^e^, Chengyuan Zhang ^a,****^, Huitang Xia ^b,c***^, Dahang Zhao ^g,**^, Feng Yuan ^a,*^*

*^a^ Department of Sports Medicine, Department of Orthopedics, Shanghai Sixth People’s Hospital Affiliated to Shanghai Jiao Tong University School of Medicine, 600 Yishan Rd, Shanghai, 200233, P.R. China*

*^b^* *Department of Plastic Surgery, The First Affiliated Hospital of Shandong First Medical University &* *Shandong Provincial Qianfoshan Hospital, 16766 Jingshi Rd., Jinan* *250014, Shandong, P.R. China*

*^c^ Jinan Clinical Research Center for Tissue Engineering Skin Regeneration and Wound Repair，Jinan, Shandong, 250014, P. R. China*

*^d^ Institute for Frontier Medical Technology, School of Chemistry and Chemical Engineering, Shanghai University of Engineering Science, 333 Longteng Rd., Shanghai 201620, P.R. China*

*^e^ Department of Orthopedics, Shanghai Public Health Clinical Center (Fudan University), 2901 Caolang Rd., Shanghai 201500, P.R. China*

*^f^ Shanghai Pengguan Biomedical Technology Co., Ltd., 2 Rd., Xinchang Town, Pudong New Area, Shanghai 200120, P.R. China*

*^g^ Department of Orthopaedics, Ruijin Hospital, Shanghai Jiaotong University School of Medicine, 197 Ruijin 2 Rd., Shanghai 200000, P.R. China*

1 These authors contributed equally to this work.

* Corresponding authors. E-mail addresses: yuanfeng19799@sina.com (F. Yuan), dahang@vip.126.com (D. Zhao), xiahuitang@163.com (H. Xia), zcy157@126.com (C. Zhang).

1. **Supplementary experimental sections**
   1. **Preparation of LDHs**

Firstly, 0.544 g NaOH was dissolved in 60 mL ultrapure water for preparing NaOH aqueous solution. Then, a mixture of 1.538 g Mg(NO_3_)_2_·6H_2_O and 0.75 g Al(NO_3_)_3_·9H_2_O was dissolved in 40 mL ultrapure water for preparing mixed salt solution. The mixed salt solution was then added dropwise to the NaOH aqueous solution, following nitrogen was introduced into the mixture. The mixture was stirred at 400 r/min for 30 min under a nitrogen atmosphere. After stirring for 30 min, the mixed solution was collected and centrifuged at 11000 r/min for 15 min. After centrifugation, the precipitate was collected. Then the precipitate was evenly dispersed in 70 mL of ultrapure water and placed in an autoclave. The autoclave was placed in a muffle furnace for 16 h and the temperature was set to 100 ℃. After heating, the solution was cooled and centrifuged (13000 r/min, 20 min) to collect the white precipitate, which was then washed with water three times.

- 1. **Water contact angle test**

The hydrophilic degree of the sample was studied by measuring the dynamic water contact angle of the scaffold at room temperature. The water contact angle of the scaffold was measured by a contact angle measuring instrument (Shanghai Zhongchen Digital Technology Equipment Co., Ltd.). PCL was dissolved and dispersed evenly in hexafluoroisopropanol solution, then poured into a cylindrical model and placed in a fume hood. After the evaporation of hexafluoroisopropanol is completed, the obtained solid cylindrical PCL bracket is used to measure the water contact angle; HA-GMA is also poured into the cylindrical model and then photocured, and the obtained HA-GMA cylindrical bracket is used to measure the water contact angle. G/P, LG/LP, and D@LG/D@LP use the same scaffold mentioned in the text to test the water contact angle.

- 1. **Mechanical performance test**

Compression test: PCL and D@LG/D@LP were compressed by high-precision pressure test machine (Shanghai Hengyu Instrument Co., Ltd.). The sample is compressed at the speed of 1 mm/min, and the compressive strength of the sample is determined by analyzing the stress-strain curve.

- 1. **Cell culture and** **preparation of osteogenic induction solution**
     1. **Cell culture**

HUVECs were cultured in DMEM medium containing 1% penicillin-streptomycin-gentamicin solution and 10% fetal bovine serum. 3T3-E1 was cultured in α-MEM medium containing 1% penicillin-streptomycin-gentamicin solution and 10% fetal bovine serum. All cells were cultured in an incubator with 37 ℃ and 5% CO_2_.

- - 1. **Preparation of osteogenic induction solution**

Osteogenic induction solution is prepared from α-MEM medium (or extract) containing10% FBS, 1% antibiotics, 0.1 μM dexamethasone, 50 μM vitamin C and 10 mM β-glycerophosphate, and the osteogenic induction solution is changed every 3 days.

1. **Supplementary figures**

**
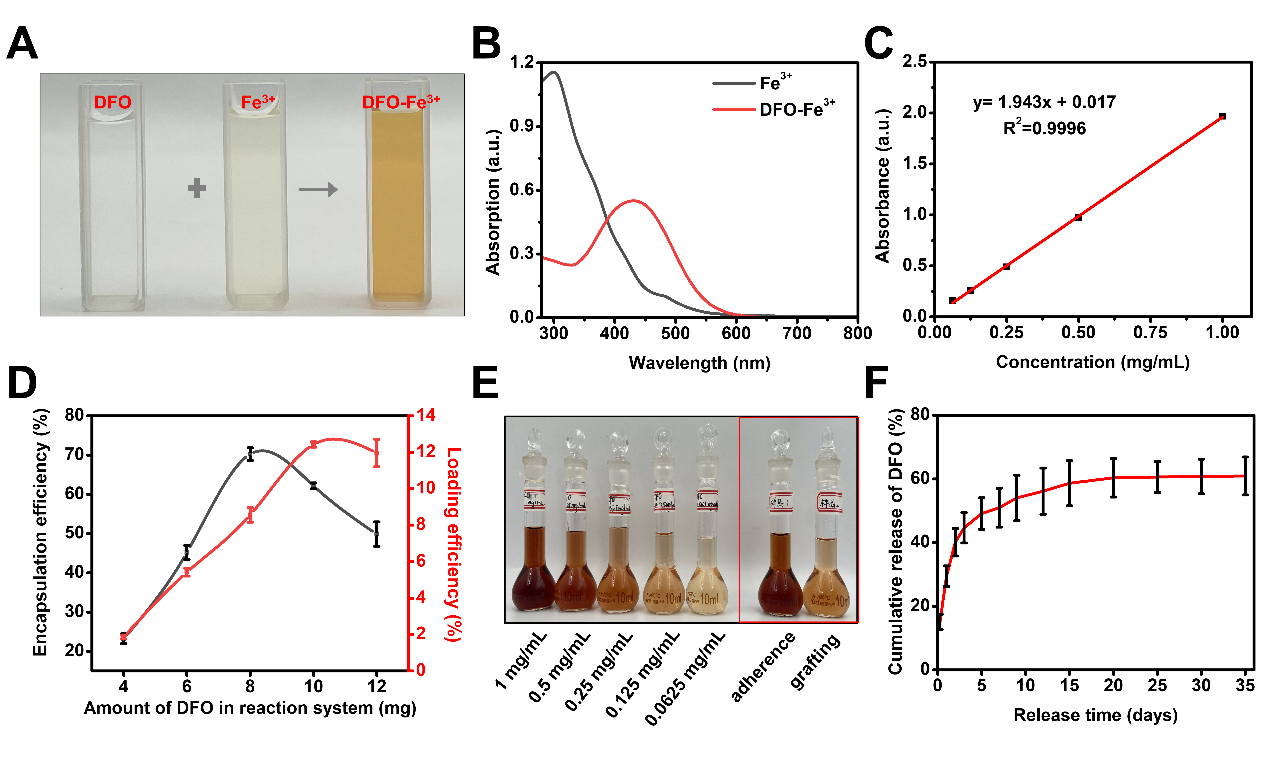
**

**Figure S1.** Characterization of DFO@LDHs. (A) DFO-Fe^3+^ complexation reaction diagram; (B) Absorbance spectrum of DFO-Fe^3+^; (C) Standard curve of DFO-Fe^3+^ solution measured at 430 nm using UV-Vis spectrophotometer; (D) Drug loading rate and encapsulation efficiency of DFO@LDHs at different DFO concentrations; (E) Colorimetric results of the supernatant after iron ion chelation in both the adhered DFO-LDHs and grafted DFO-LDHs reaction systems; (F) DFO drug release kinetics of DFO@LDHs.

**
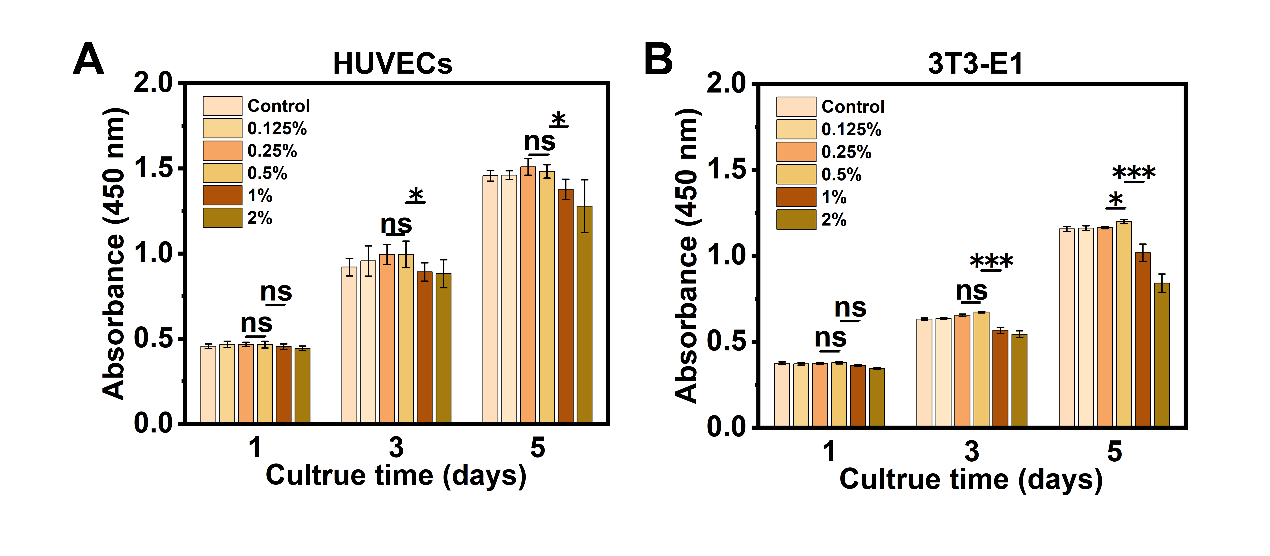
**

**Figure S2.** Optimal drug loading concentration of D@LG/D@LP composite scaffolds. (A) HUVECs proliferation after 1, 3, and 5 days of culture; (B) 3T3-E1 proliferation after 1, 3, and 5 days of culture. (n = 6; *p < 0.05, **p < 0.01, and ***p < 0.001, and “ns” indicated no significant difference).


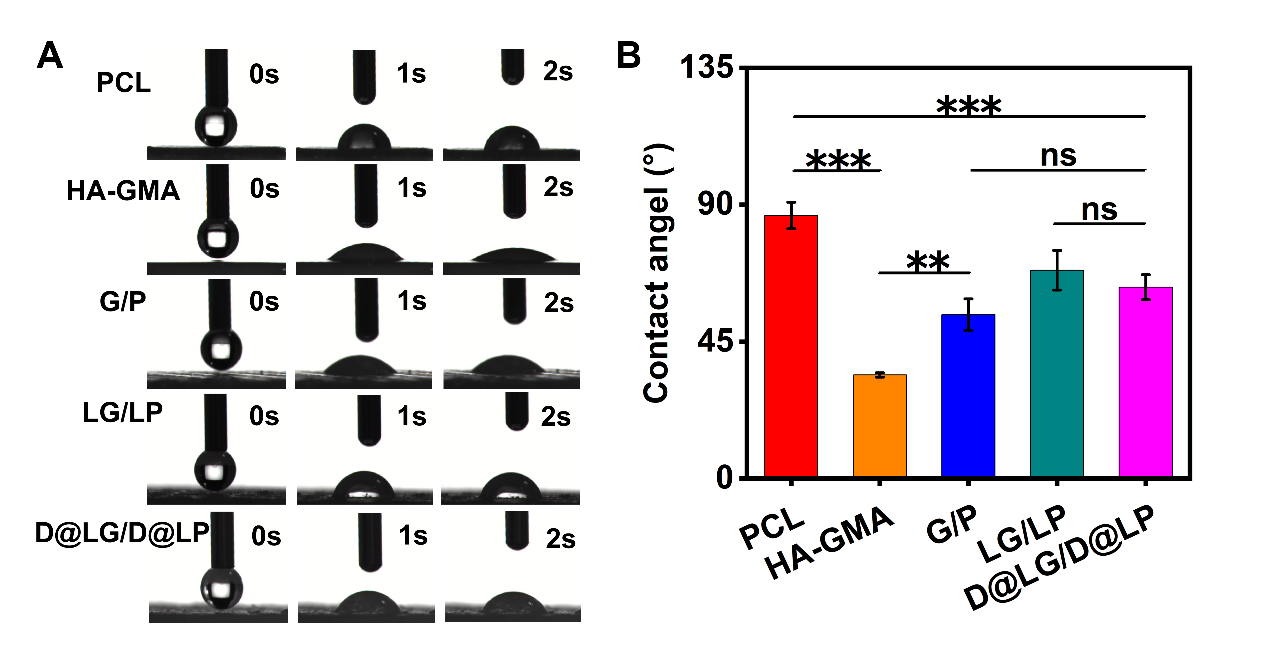


**Figure S3.** Water contact angle. (A) Dynamic water contact angle observation of PCL, HA-GMA and G/P, LG/LP, and D@LG/D@LP and (B) data statistical histograms. (n = 3; *p < 0.05, **p < 0.01, and ***p < 0.001, and “ns” indicated no significant difference).


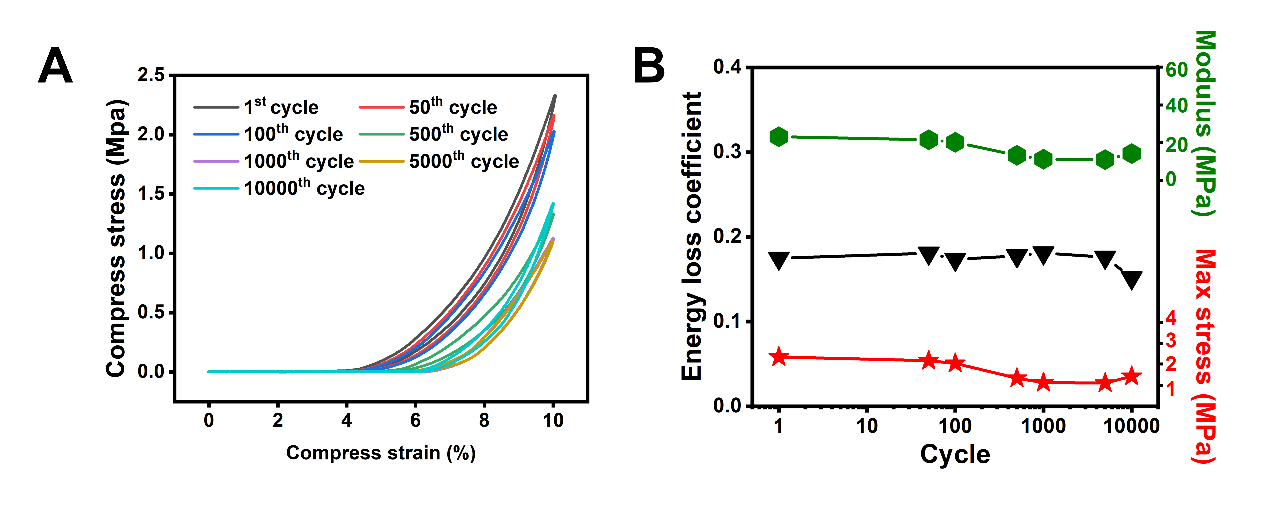


**Figure S4.** Mechanical properties of PCL. (A) Compression curves of PCL scaffold under 10000 cycles; (B) Storage modulus, energy loss coefficient, and max stress of PCL scaffold at 10000-cycle compression.

**
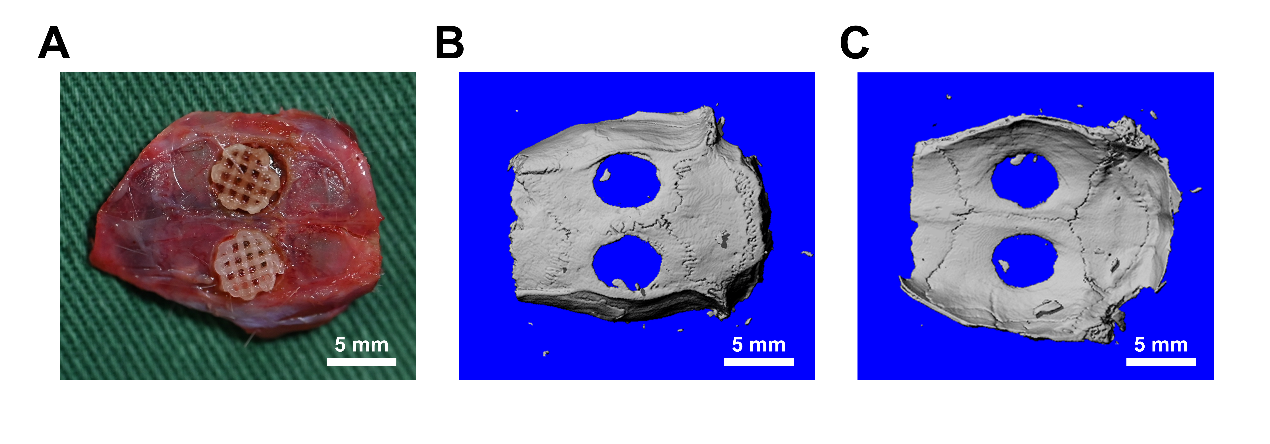
**

**Figure S5.** Characterization of the critical-sized cranial defect model at Day 0. (A) Macroscopic photograph of the rat calvarium immediately after defect creation, showing a standardized circular defect; (B) Top view and (C) Bottom view of the micro-CT 3D reconstruction of the defect site at Day 0, confirming the complete removal of the parietal bone and the consistency of the initial defect size.

**Table S1. MIP data results**

| **Intrusion Data Summary** | **PCL** | **G/P** | **LG/LP** | **D@LG/**  **D@LP** | **Unit** |
| --- | --- | --- | --- | --- | --- |
| Total intrusion volume | 0.0921 | 0.4446 | 0.4830 | 0.7875 | mL/g |
| Total pore area | 13.484 | 14.441 | 20.131 | 17.798 | m^2^/g |
| Median pore diameter (volume) | 107355.54 | 41188.79 | 54215.01 | 57163.97 | nm |
| Median pore diameter (area) | 5.71 | 5.26 | 6.91 | 12.66 | nm |
| Average pore diameter (4V/A) | 27.33 | 123.15 | 95.98 | 176.99 | nm |
| Bulk density | 1.0881 | 0.8222 | 0.7674 | 06243 | g/mL |
| Apparent (skeletal) density | 1.2093 | 1.2959 | 1.2195 | 1.2281 | g/mL |
| Porosity | 10.0252 | 36.5552 | 37.0680 | 49.1654 | % |
| Stem Volume Used | 4 | 20 | 24 | 19 | % |

**Table S2.** **Diffraction angle and plane spacing data of PCL, G/P, LG/LP and D@LG/D@LP from XRD analysis.**

| **Diffraction plane (*hkl*)** | **2θ.peak position (°)** | | | | **Plane spacing (d,·Ã)^a^** | | | |
| --- | --- | --- | --- | --- | --- | --- | --- | --- |
|  | **PCL** | **G/P** | **LG/LP** | **D@LG**  **/D@LP** | **PCL** | **G/P** | **LG/LP** | **D@LG**  **/D@LP** |
| -(611) | 21.44 | 21.32 | 21.16 | 21.38 | 4.14 | 4.16 | 4.19 | 4.15 |
| -(802) | 22.02 | 21.88 | 21.86 | 22.06 | 4.03 | 4.05 | 4.06 | 4.02 |
| (2400) | 23.52 | 23.62 | 23.61 | 23.78 | 3.79 | 3.77 | 3.77 | 3.74 |
| (3000) | 29.78 | 29.77 | 29.81 | 29.78 | 3 | 3 | 2.99 | 3 |
| (2312) | 36.1 | 35.88 | 36.12 | 36.2 | 2.49 | 2.51 | 2.49 | 2.48 |
| (921) | 38.4 | 38.36 | 38.12 | 36.4 | 2.35 | 2.35 | 2.37 | 2.47 |
| -(1222) | 43.6 | 43.48 | 43.32 | 43.54 | 2.08 | 2.08 | 2.09 | 2.08 |

^a^ The plane spacing of different diffraction planes (*d_hkl_*) was calculated from the Bragg’s Law:

*d_hkl_*$=\frac{\lambda}{2\sin\theta}$

Where $\lambda$ is the wavelength of the copper anode source ($\lambda$ = 1.54 Å) and $\theta$ stands for the diffraction angle of each indexed diffraction plane.
